# Dynamics and stabilization mechanism of mitochondrial cristae morphofunction associated with turgor-driven cardiolipin biosynthesis under salt stress conditions

**DOI:** 10.1038/s41598-022-14164-3

**Published:** 2022-07-01

**Authors:** Keisuke Nakata, Yuto Hatakeyama, Rosa Erra-Balsells, Hiroshi Nonami, Hiroshi Wada

**Affiliations:** 1 The United Graduate School of Agricultural Science, Ehime University, Matsuyama, Ehime Japan; 2 Graduate School of Agriculture, Ehime University, Matsuyama, Ehime Japan; 3 Department of Organic Chemistry and CIHIDECAR-CONICET, University of Buenos Aires, Buenos Aires, Argentina

**Keywords:** Plant physiology, Plant stress responses

## Abstract

Maintaining energy production efficiency is of vital importance to plants growing under changing environments. Cardiolipin localized in the inner mitochondrial membrane plays various important roles in mitochondrial function and its activity, although the regulation of mitochondrial morphology to various stress conditions remains obscure, particularly in the context of changes in cellular water relations and metabolisms. By combining single-cell metabolomics with transmission electron microscopy, we have investigated the adaptation mechanism in tomato trichome stalk cells at moderate salt stress to determine the kinetics of cellular parameters and metabolisms. We have found that turgor loss occurred just after the stress conditions, followed by the contrasting volumetric changes in mitochondria and cells, the accumulation of TCA cycle-related metabolites at osmotic adjustment, and a temporal increase in cardiolipin concentration, resulting in a reversible topological modification in the tubulo-vesicular cristae. Because all of these cellular events were dynamically observed in the same single-cells without causing any disturbance for redox states and cytoplasmic streaming, we conclude that turgor pressure might play a regulatory role in the mitochondrial morphological switch throughout the temporal activation of cardiolipin biosynthesis, which sustains mitochondrial respiration and energy conversion even under the salt stress conditions.

## Introduction

Stabilizing the efficiency of energy production is vital to the survival of all non-motile organisms living in various stress environments. Mitochondria play an essential role in cellular metabolisms by providing adenosine 5'-triphosphate (ATP) due to oxidative phosphorylation, sustaining the velocity of cytoplasmic streaming in the cells. In mitochondria, energy production occurs in the inner mitochondrial membrane (IMM) containing phospholipids involved in the chemiosmotic coupling, establishing a transmembrane proton gradient by activation of ATP synthase. While most studies have been long postulating chemiosmotic coupling in the classical infolding model with flat cristae^1^, as known as ‘baffle model’, electron tomography has provided another model, called ‘cristae junction (CJ) model’, in which the shape of cristae is contrastive and narrow tubular openings, CJs are connected with the intermembrane space, different from the infolding model that exhibits relatively large openings connecting the intercristal space to the intermembrane space^2,3^. A growing body of evidence is accumulating to demonstrate that CJ model with tubulo-vesicular crista structure also holds true and is common for diverse eukaryotes as well as the infolding model^4^.

Also, the knowledge of the regulation and function of mitochondria has been expanded due to the understanding for the roles of unique phospholipid cardiolipin (CL) localized in the IMM^5^. CL is a diphosphatidylglycerol lipid in which two phosphatidic acid (PA) moieties are connected with a central glycerol backbone to form a peculiar structure^6^. It participates in the IMM curvature formation to topologically modify the crista structure (see detailed structure shown in Fig. S1), which is required for the optimization of respiratory complexes and ATP synthase under stress conditions^7^. In bacterial cells, phospholipid synthesis is influenced by cellular volumetric changes to the altered external osmotic pressure^8,9^. Several researchers have shown an increase in CL concentration through the osmoregulatory process in response to salt stress^8,9^, implying the involvement of turgor pressure (Ψ_p_). However, possible role(s) of Ψ_p_ on mitochondria morphology and CL synthesis remains obscure.

In general, Ψ_p_ plays various fundamental roles in structural integrity, development, and other physiological functions including stress responses. In plants, changes in Ψ_p_ propagate inside cells and/or tissues hydrostatically at the speed of sound^10^. External application of 0.1–0.2 MPa of pressure pulse into the cells causes plasmodesmatal closure^11^ and inhibition of aquaporins (AQPs) that facilitate water flow across membranes^12^. It has been also reported that changes in Ψ_p_ might be involved in the regulation of nuclear gene expression at dehydration^13,14^, abscisic acid (ABA) biosynthesis^15^, and other processes (e.g., fruit softening^16^; starch phosphorylation^17^). Similar mechanism(s) may exist in plant mitochondrial energy production at salt stress. Simultaneous measurement of Ψ_p_ and metabolites in the single-cells would be required to address the above question.

During the past decade, metabolomics at ambient pressure has been dramatically extended (e.g., ESI-based methods^18,19^). Various ambient mass spectrometry (MS) techniques have been applied to the cell metabolomics under ambient and open-air conditions^20–22^. Among these, the analytical method sampling cellular fluids by using a ‘transparent’ microcapillary inspired single-cell ambient MS^21^. A cell pressure-probe (CPP)^23^, known as the device allowing direct cell Ψ_p_ determination in growing plants using a finely tapered transparent microcapillary, was combined with Orbitrap mass spectrometer to develop an in situ single-cell analytical method^24^. In the current method, termed “picolitre pressure-probe electrospray-ionization mass spectrometry (picoPPESI-MS)”, an internal electrode and ionic liquid solution have been adopted to achieve high resolution and sensitivity in the MS analysis, retaining the function of the CPP with pressure and picolitre volume control (Fig. S2)^25^.

PicoPPESI-MS analysis can be minimally invasively carried out after the direct determination of cell hydraulic properties, such as Ψ_p_ and hydraulic conductivity in the cells in the growing plants, conceptually different from other analytical methods with a non-transparent pipette or a needle. It was shown that picoPPESI-MS was capable for discriminating metabolites between the neighboring cells^25,26^ as well as at single rice pollen grains enclosed in the growing anthers in the attached plants^27^. As shown previously^28^, this approach also allows real-time investigation of the inherent heterogeneity in single-cells, providing *in-situ* biological information including the cell water status that had never been attainable in the past. Moreover, using a ‘transparent’ microcapillary allows to determine the volume of fluids by the mean of pressure control. In picoPPESI-MS, cell sap collection can be completed within 0.5 s, quicker than the half time (typically, > 2 s) for water permeability in plant cells^29,30^, implying that potential dilution and contamination effects caused by water and/or solute movements between the impaled cells and neighboring cells might be negligible. While a snap-shot picoPPESI-MS analysis has been succeeded in various plant cells^25–28,31^, no attempt has been made for the time-course picoPPESI-MS analysis coupled with the volume determination.

Here, we have studied the cellular adaptation mechanism at salt stress from the viewpoint of cell water relations. Adopting the single stalk cells of type II trichome in intact tomato plants as a model system, we applied the plants to the relatively small extent of salt stress conditions. Collecting the equivalent volume of cellular fluids from the target single-cells in time-course picoPPESI-MS analysis should provide a more precise single-cell metabolomics. Conducting the time-course picoPPESI-MS and cytoplasmic streaming analyses in conjunction with transmission electron microscopy (TEM), dynamics for Ψ_p_-induced CL biosynthesis has been revealed in the tubulo-vesicular cristae of tomato plants. Based on the kinetics of cellular parameters and metabolisms, the dynamic adaptation mechanism to salt stress will be discussed in terms of mitochondrial cristae morphofunctional modification.

## Results

### Cell water status and cytoplasmic streaming velocity at salt stress

**Figure 1:**
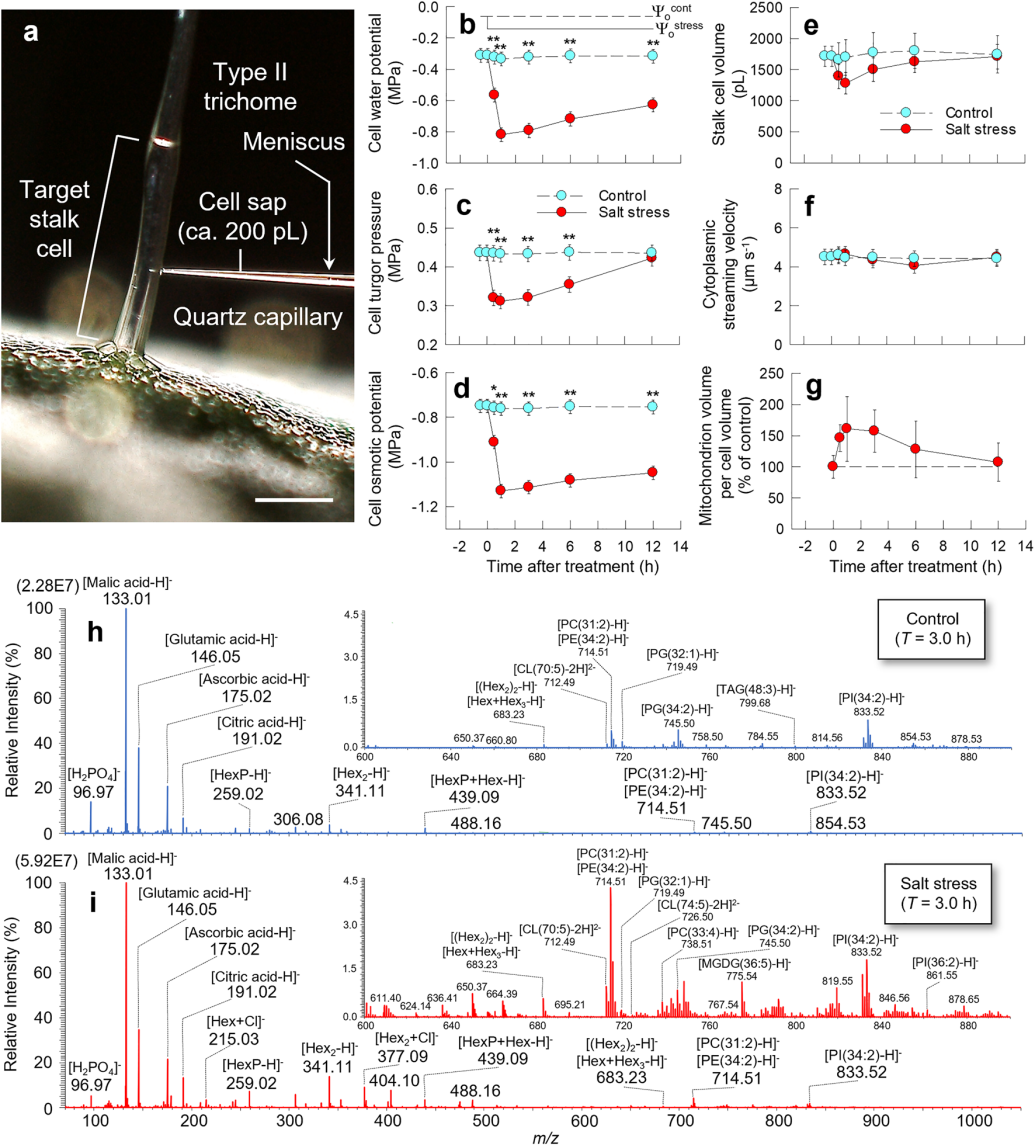
Time-course of changes in cellular parameters and picoPPESI negative ion mode mass spectra in tomato trichome stalk cells under salt stress conditions. An image of tip insertion into the stalk cells (**a**). Changes in the media water potential and cell water potential (Ψ_w_) (**b**), cell turgor (Ψ_p_) (**c**), cell osmotic potential (Ψ_s_) (**d**), cell volume (*V*_*o*_) (**e**), cytoplasmic streaming velocity (**f**), mitochondrion volume per the cell volume (*V*_*Mito*_/*V*_*o*_) (**g**) under salt stress conditions. PicoPPESI negative ion mode mass spectra obtained from the stalk cells under control and salt stress conditions at 3 h (**h** and **i**). Cyan and red plots indicate control and salt stress treatment, respectively. Bar = 200 μm. For Ψ_w_, Ψ_p_, Ψ_s_, and cytoplasmic streaming velocity, data are means ± SE for 3–32 cells from 3 to 6 plants in each treatment (**b**, **c**, **d**, and **f**). (**e**) The *V*_*o*_ data are means ± SE for 11–16 cells from 3 to 6 plants in each treatment. (**g**) The *V*_*Mito*_/*V*_*o*_ data is means ± SE for 5–12 cells from 3 to 6 plants in each treatment. Significant difference at the 0.01 and 0.001 probability levels by *t*-test is indicated with * and **, respectively. The data represent repeated experiments with 8–32 stalk cells in total from 3 to 6 plants in each treatment (**h** and **i**). Hex: hexose; HexP: hexose phosphate; CL: cardiolipin; PC: phosphatidylcholine; PE: phosphatidylethanolamine; PI: phosphatidylinositol; PG: phosphatidylglycerol; TAG: triacylglycerol; PS: phosphatidylserine; MGDG: monogalactosyl diacylglycerol. Line graphs were created with Sigmaplot 13.0.

Under the salt stress conditions (the media water potential, Ψ_o_^stress^ =  − 0.14 MPa), the transpiration rate and leaf water status (i.e., Ψ_p_, osmotic potential (Ψ_s_), and water potential (Ψ_w_) determined at tissue level) remained to be constant (Fig. S3), whereas the water status of target stalk cells was altered dramatically (Fig. 1a–d). After the initiation of treatment, stalk cell Ψ_p_ considerably decreased, reaching 0.312 ± 0.002 MPa (Mean ± SE, *n* = 15, CV = 2.54%) at 0.5 h (*p* < 0.001, Fig. 1c), whereas there were no changes in cytoplasmic streaming velocity in the same cells throughout the treatment (Fig. 1b–d and f). A reduction in *V*_*o*_ was accompanied with Ψ_p_ loss, and contrastingly a transient increase in mitochondrion volume per cell volume (*V*_*Mito*_/*V*_*o*_) was observed (Fig. 1c, e, and f). And thereafter, cell Ψ_p_ started to increase at osmotic adjustment and recovered in 12 h (Fig. 1c).

### Cell metabolisms including phospholipid synthesis at salt stress

After videotaping the cytoplasmic streaming followed by in situ Ψ_p_ assay, oil pressure was immediately reduced down to subzero pressure, and ca. 200 pL, corresponding to 11.4–15.4% of cell volume (*V*_*o*_) of cellular fluids, was collected in the microcapillary tips in all runs by means of pressure control (see Methods) (Fig. 1a). By applying a high voltage (− 4 kV) in picoPPESI-MS operated in negative ion mode, metabolites contained in the equivalent volume as gas ions were directly introduced into the Orbitrap mass spectrometer and detected as deprotonated [M − H]^−^ and/or [M + Cl]^−^ species (M: molecular weight) as well as deprotonated [M’ − H]^−^ (M’: cluster weight). These clusters are the stable molecular aggregates formed both in the solution and during the ESI process because the intermolecular interactions among the molecular unities are quite strong (e.g., hydrogen bridge); its formation highly depends on the concentration of molecular constituents in the analyzed solution; at higher concentrations, the chance to obtain cluster signals in ESI–MS increases^32^.

In the picoPPESI mass spectra, the peaks of metabolites related to glycolysis (e.g., hexose (Hex) (*m/z* 179), hexose phosphate (HexP) (*m/z* 259), Hex_2_ (*m/z* 341 and 377)), tricarboxylic acid (TCA) cycle (e.g., malate (*m/z* 133) and citrate (*m/z* 191)), amino acids (e.g., glutamate (*m/z* 146)), cluster ions (e.g., [HexP + Hex − H]^−^ (*m/z* 439), [Quinic acid + Hex − H]^−^ (*m/z* 533), [(Hex_2_)_2_ − H]^−^ (*m/z* 683), [Hex + Hex_3_ − H]^−^ (*m/z* 683)), and a vast number of lipid metabolite ion peaks (e.g., [CL(70:5) − 2H]^2−^ (*m/z* 712), [Phosphatidylcholine; PC(31:2) − H]^−^ (*m/z* 714), [Phosphatidylethanolamine; PE(34:2) − H]^−^ (*m/z* 714), [phosphatidylglycerol; PG(32:1) − H]^−^ (*m/z* 719), [CL(74:5) − 2H]^2−^ (*m/z* 726), [PC(33:4) − H]^−^ (*m/z* 738), [PG(34:2) − H]^−^ (*m/z* 745), [Phosphatidylserine; PS(33:0) − H]^−^ (*m/z* 748), [Monogalactosyl diacylglycerol; MGDG(36:5) − H]^−^ (*m/z* 775), [Phosphatidylinositol; PI(34:2) − H]^−^ (*m/z* 833), and [PI(36:2) − H]^−^ (*m/z* 861)) were detected as major ions (Fig. 1h and i). Regarding the identification of HexP (*m/z* 259), Glc6P, Fru6P, and Glc1P standard solutions were prepared, and each solution was individually analyzed in the negative ion mode under the same experimental conditions (Fig. S4a–c). The three theoretical fragment ions (*m/z* 138, 168, and 199) originated from HexP have been simultaneously observed in the picoPPESI mass spectra of both each of the Glc6P standard solution and the cell sap (Figs. S4a, S5-1, and S5-2), suggesting that *m/z* 259 signal observed in cell sap would be assigned as mostly the carbohydrate Glc6P.

Fig. S6 shows the relationship between the number of moles and corresponding signal intensity of each standard phosphorous-containing metabolite solution ([PO_3_]^−^, [H_2_PO_4_]^−^, [Glc6P − H]^−^, [Glc1P − H]^−^, [Fru6P − H]^−^, and [ATP − 2H]^2−^). For each metabolite, the dynamic range (i.e., *y*-axis) shown in Fig. S6 was highly correlated with the concentration of each standard solution.

**Figure 2:**
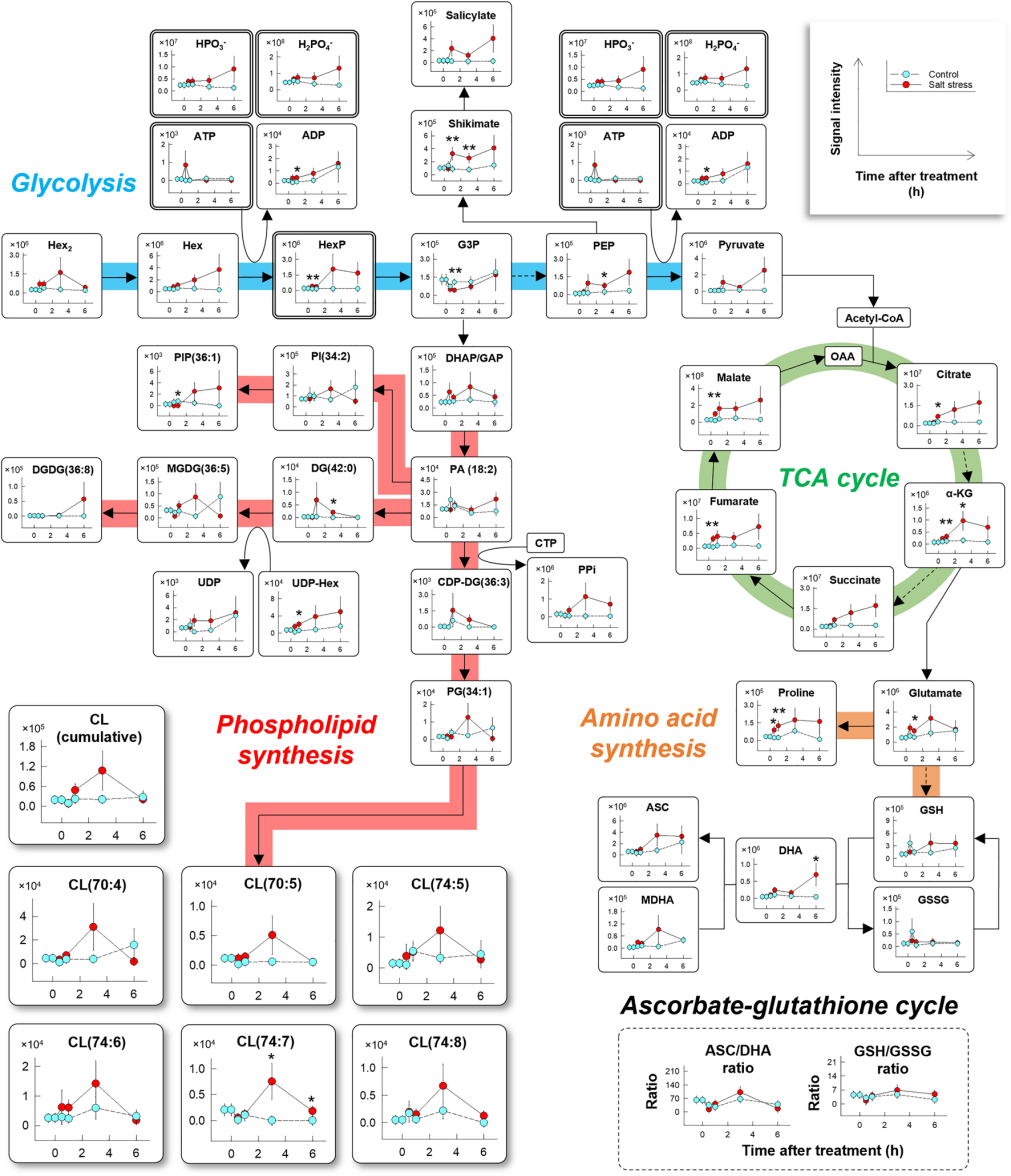
Metabolic network altered in the stalk cells under salt stress conditions. The signal intensity in picoPPESI negative ion mode mass spectra plotted against the time after treatment (h). Ascorbate (ASC)/dehydroascorbate (DHA) and glutathione (GSH)/oxidized glutathione (GSSG) ratios calculated are also shown. Cyan and red plots indicate control and salt stress treatment, respectively. Data are means ± SE for 8–32 stalk cells from 3 to 6 plants in each treatment. The *p*-value at the 0.1 and 0.05 probability levels indicated with * and **, determined using *t-*test. Hex: hexose; HexP: hexose phosphate; ATP: adenosine 5’-triphosphate; ADP: adenosine 5’-diphosphate; G3P: glycerol-3-phosphate; DHAP: dihydroxyacetone phosphate; GAP: glyceraldehyde 3-phosphate; PEP: phosphoenolpyruvate; α-KG: α- ketoglutarate; OAA: oxaloacetate; MDHA: monodehydroascorbic acid; PA: phosphatidic acid; DG: diacylglycerol; UDP: uridine 5’-diphosphate; MGDG: monogalactosyl diacylglycerol; DGDG: digalactosyl diacylglycerol; CTP: cytidine triphosphate; PPi: bisphosphate; CDP-DG: cytidine diphosphate diacylglycerol; PI: phosphatidylinositol; PIP: phosphatidylinositol monophosphate; PG: phosphatidylglycerol; CL: cardiolipin. Line graphs were created with Sigmaplot 13.0.

Changes in glycolysis, TCA cycle, and phospholipid synthesis-related metabolites were dynamic under the stress conditions (Fig. 2). The content of these metabolites overall increased after imposing the salt stress (Fig. 2 and Table S1). A transient increase in ATP signal synchronized with a reduction in the content of glycerol-3-phosphate (G3P) and phosphatidylinositol monophosphate (PIP)(36:1) has been detected at 0.5 h after treatment (Fig. 2) together with some minor fragments from organophosphate metabolites observed during the picoPPESI process (see Figs. S4, S5-1, S5-2, and S7 for Glc6P, Fru6P, Glc1P, and ATP, respectively). The concentration of phosphoenolpyruvate (PEP), pyruvate, salicylate, shikimate, bisphosphate (PPi), and several phospholipid metabolites including dihydroxyacetone phosphate (DHAP)/glyceraldehyde 3-phosphate (GAP), diacylglycerol (DG)(42:0), and cytidine diphosphate diacylglycerol (CDP-DG)(36:3) substantially increased at 1 h and thereafter maintained at high concentration (Fig. 2). The signal intensity of adenosine 5’-diphosphate (ADP), Hex, [PO_3_]^−^, [H_2_PO_4_]^−^, uridine 5’-diphosphate (UDP), and UDP-Hex, most TCA-related metabolites and amino acids gradually increase over time (Fig. 2 and Table S1). The Hex_2_ concentration declined between 3 and 6 h in contrast with the pattern of Hex concentration (Fig. 2). A series of CLs exhibiting strong signals have also been detected in the picoPPESI mass spectra, particularly CL(70:4), CL(70:5), CL(74:5), CL(74:6), CL(74:7), and CL(74:8) (Figs. 2 and S8 and Table S1). Of these, the concentration of CL(70:4) shows that this metabolite is the major CL. Furthermore, the increase in these CLs synchronized with that of the intermediate metabolites (PA, PG as CL precursors, and CDP-DG) (see PG(34:1) and DHAP/GAP as the corresponding metabolites shown in Figs. 2 and S8 and Table S1 for other intermediates). For the major CL, CL(70:4) was considered to be composed of either PG(34:1) and PG(36:3) or PG(34:2) and PG(36:2), or both (Fig. S8 and Table S1). Throughout the experiment, there were no treatment differences in both ratios of ascorbate (ASC)/dehydroascorbate (DHA) and glutathione (GSH)/oxidized glutathione (GSSG) (Fig. 2) simultaneously determined in the same cells.

### Mitochondrial morphological changes at salt stress

**Figure 3:**
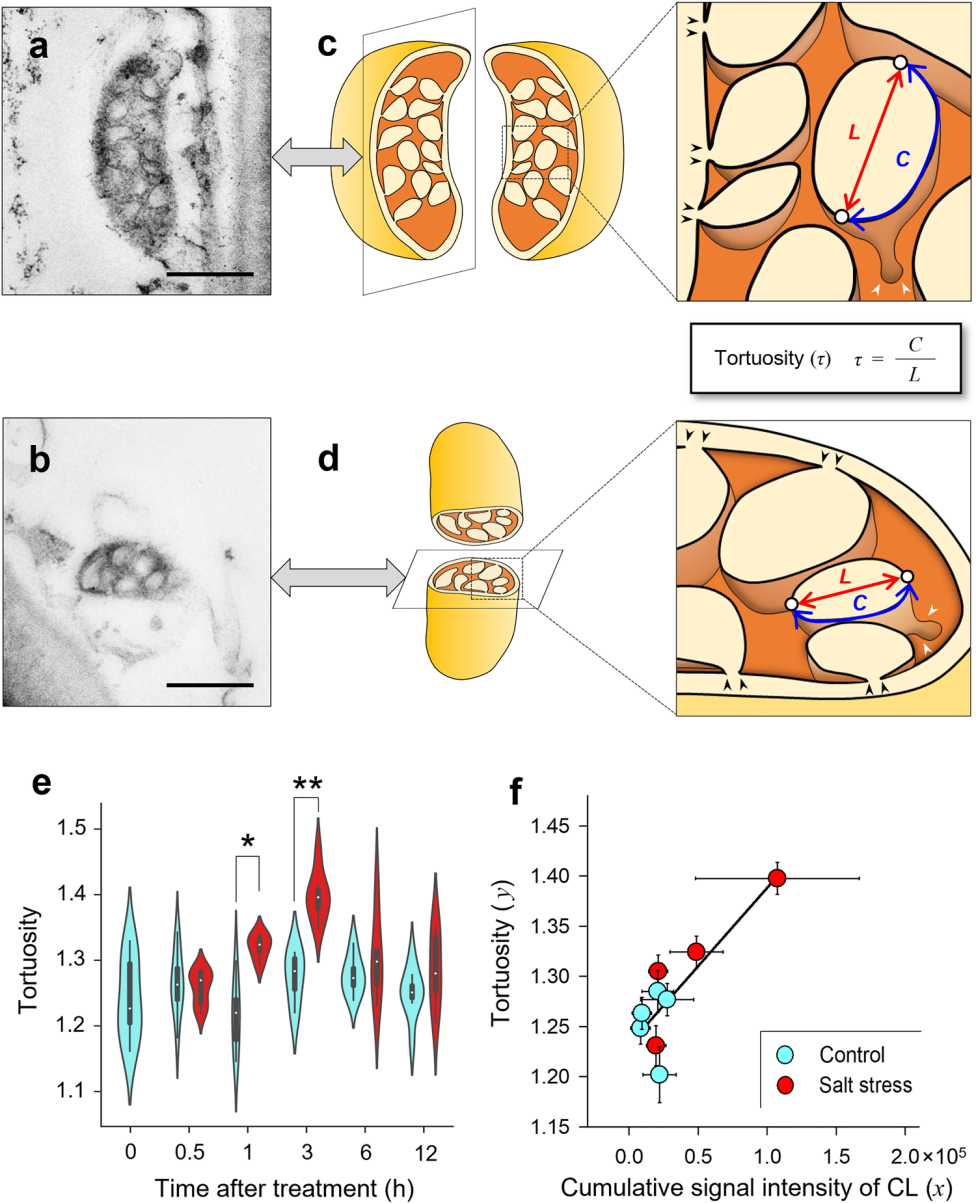
Morphological changes in the mitochondrial internal structures of tomato trichome stalk cells (see Methods) under salt stress conditions. Longitudinal (**a**) and transverse (**b**) sections of TEM images and their diagrams of mitochondrial internal structures with cristae junction model in the cells (**c** and **d**). In **c** and **d**, each expanded image shows the putative structure of tubulo-vesicular cristae at each section. Time-course of changes in tortuosity (*τ*) of the cristae determined by using an image analysis throughout the treatment (**e**), and *τ* plotted against the cumulative CL signal intensity from Fig. 2**f**. Bars = 500 nm (**a** and **b**). In **e** and **f**, cyan and red plots indicate control and salt stress treatment, respectively. Data were obtained from 94 to 315 cristae in 16–44 mitochondria in 3–6 plants in each treatment. Significant difference at the 0.01 and 0.001 probability levels by *t*-test is indicated with * and **, respectively. Regression of *τ* and cumulative signal intensity of cardiolipin (CL) (**f**). Data are means ± SEs for 8–32 stalk cells from 3 to 6 plants. The regression line between the cumulative signal intensity of CL (*x*) and *τ* (*y*) in CL is* y* = − 0.16 × 10^−5^*x* + 1.23, with R^2^ = 0.70 (*p* < 0.005). A violin plot was created with Python 3.9.1. Creating a scatter plot and the linear regression were performed in SigmaPlot 13.0.

In addition to the picoPPESI-MS analysis, the collected cell sap was used for TEM observation. When an aliquot of cell sap was loaded onto the TEM grid mesh and viewed with a TEM, numerous mitochondria- and membrane-like structures were observed (Fig. S9). When the fixed cell samples were observed by using the TEM (Figs. 3, S10–12), a nucleus, a central vacuole, mitochondria, plastids, and peroxisomes were identified in the cells; the vacuoles were observed to be the major organelle, occupying approximately 75% of the cells (74.6 ± 3.0%, Mean ± SE, *n* = 24, CV = 19.4%), which corresponds to 98% of cytosol (Table S2). Besides vacuoles, mitochondria with many tubulo-vesicular cristae were most frequently observed as the major organelle in the cytosol, and their contribution percentage in the cytosol was ca. 51% (51.0 ± 8.6%, Mean ± SE, *n* = 24, CV = 79.7%) (Table S2). Mitochondrion volume (*V*_*Mito*_), number of mitochondria per cell, and total *V*_*Mito*_/*V*_*o*_ were determined overtime during the experimental duration (Fig. S10), and *V*_*Mito*_ was shown to increase from 0.5 to 6 h, but with no significant difference, regardless of the temporal reduction in *V*_*Mito*_ (Figs. 1e, g, and S10a), as described above. Although there was no significant difference in the number of mitochondria per cell (Fig. S10b), an increase in total *V*_*Mito*_/*V*_*o*_ due to the increase in *V*_*Mito*_ have been transiently observed, which synchronized with a reduction in *V*_*o*_ (Figs. 1e, g, and S10c).

When the internal structures of mitochondria in the cells were closely inspected using the TEM, similar ultrastructure with the CJ model generally accepted^3^ were observed (Fig. 3a–d). In each treatment, morphological changes in the internal structures of the mitochondria have been evaluated by determining the intracrista space area, the number of cristae per mitochondrion, interspace area per mitochondrion, crista tortuosity (*τ*), and crista apex angle (*θ*)^33^ throughout treatment (Figs. 3, S10d–f, S11, and S12). Although there was no treatment difference in the intracrista space area, the number of cristae per mitochondrion, the interspace area per mitochondrion during the treatment (Fig. S10d–f), the violin plot based on the results of image analysis indicates that different from control, the proportion of cristae structures with high *τ* started to increase at 1 h after the treatment, reaching to the maximum value at 3 h (*n* in control and salt stress treatment = 244 and 315, respectively). Simultaneously, *θ* declined only at 3 h after the treatment (Fig. S12c). These parameters were shown to be reversible, as the *τ* and *θ* returned to the pretreatment values between 6 and 12 h after the treatment (Figs. 3e and S12c). The data also showed that the signal intensity of each CL and cumulative CL directly determined by picoPPESI-MS were highly correlated with both *τ* and *θ* of the mitochondria in the cells (Figs. 3f, S12d, and S13).

### The kinetics of cellular parameters and metabolisms at salt stress

**Figure 4:**
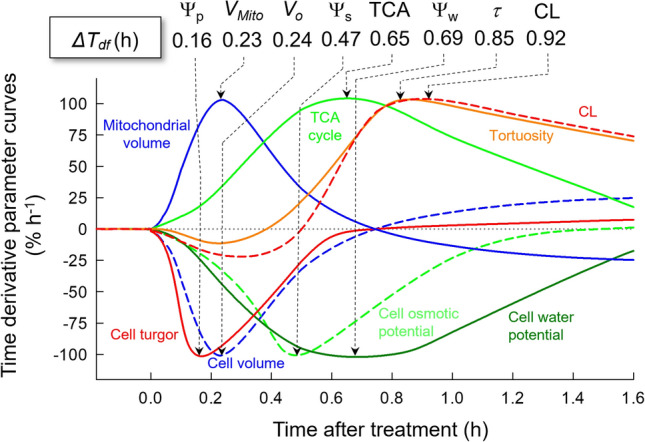
Kinetics of cellular parameters on turgor-driven cardiolipin (CL) biosynthesis and mitochondrial membrane dynamics in the stalk cells at salt stress. Time derivative parameter curves of cell turgor (Ψ_p_, red line), mitochondrial volume (*V*_*Mito*_, blue line), cell volume (*V*_*o*_, blue dashed line), cell osmotic potential (Ψ_s_, lime green dashed line), cumulative signal intensity of tricarboxylic acid (TCA) cycle metabolites (lime green line), cell water potential (Ψ_w_, green line), crista tortuosity (*τ*, orange line), and CL (red dashed line) (also see Fig. S14A). All parameters were scaled by setting the maximum/minimum absolute value of the change to be + 100/− 100%, and then the time derivative parameter curves were generated. *ΔT*_*df*_ indicates the time required for reaching the maximum/minimum rate after the treatment for each parameter. The graph was created with Sigmaplot 13.0.

The time derivative parameter curves for Ψ_w_, Ψ_p_, Ψ_s_, *V*_*o*_, *V*_*Mito*_, TCA cycle (detected as the cumulative signal intensity of TCA-related metabolites), CL (cumulative CL signal intensity), and *τ* were generated with the above data (Fig. S14a). When the time (*ΔT*_*df*_) required for reaching the maximum (+ 100%)/minimum (− 100%) rate after the treatment in each parameter was determined, *ΔT*_*df*_ for Ψ_p_, *V*_*Mito*_, *V*_*o*_, Ψ_s_, TCA cycle, Ψ_w_, *τ*, and CL was estimated to be 0.16, 0.23, 0.24, 0.47, 0.65, 0.69, 0.85, and 0.92 h, respectively (Fig. 4).

## Discussion

In this study, we have used a picoPPESI-MS-based cell metabolomics to analyze the effect of salt stress in tomato trichome single-cells. Combining time-course single-cell analyses with TEM observations in the cells, in situ cellular responses were measured in real-time during the stress duration and the kinetics of each cellular parameter has been determined (Figs. 4 and S14a). The data also show that tomato mitochondria were composed of multiple tubulo-vesicular cristae (i.e., CJ model). After the imposition of moderate salt stress (i.e., Ψ_w_ = − 0.14 MPa), cellular and organelle responses were found to be dynamic, in contrast with the tissue responses (see leaf water status and transpiration rate in Fig. S2). Ψ_p_ loss occurred as the earliest event in the cells, followed by a transient increase in *V*_*Mito*_ synchronized with a reduction in *V*_*o*_, lowering of Ψ_s_ (Fig. 1b–d), accumulation of TCA-related metabolites, a reduction in cell Ψ_w_, and IMM morphological changes (see the altered *τ* and *θ* in Figs. 3 and S12) due to the temporally enhanced CL biosynthesis (Figs. 2, 3e, and 4). It is noted that none of the cellular redox states and cytoplasmic streaming velocity were altered throughout the treatment (Figs. 1, 2, and S14b–d). By utilizing the picoPPESI-MS that retains the function of CPP, the volume of cellular fluids collected was adjusted in all runs (see Results), which gave a more precise single-cell metabolomics immediately after Ψ_p_ determination in the ‘same single-cells’ (see Fig. 1). Because Ψ_p_ loss preceded CL biosynthesis that is responsible for the observed cristae morphofunctional changes, it is proposed that changes in Ψ_p_ may serve as a hydraulic signal on stabilizing mitochondrial function through the CL accumulation at salt stress.

In this study, we have subjected the small extent of stress conditions to the root system in tomato plants. If somewhat larger extent of stress than that applied here were given, changes in each parameter would be completed within minutes, which is too fast to address the kinetics even using picoPPESI-MS, as this method typically requires 3 min per run (see Materials and Methods). Also, the treatment difference in media water potential (*Δ*Ψ_o_ = 0.09 MPa) was set to be smaller than 0.2 MPa, above which hydraulic disturbances are known to occur (plasmodesmata closure^11^; AQP closure^12^). Choosing the moderate extent of stress to perform a series of time-course analyses in the same cells allows us to determine the kinetics of changes in cellular events including CL metabolism without any disturbance of cytoplasmic streaming and cellular redox states, at least for ASC/DHA and GSH/GSSG ratios (Figs. 1, 2, 4, and S14) and illustrate the possible role of Ψ_p_ on the enhancement of primary metabolism and phospholipid synthesis to sustain the mitochondrial energy efficiency even at salt stress.

Cytoplasmic streaming occurs as myosin-linked organelles that move along actin filaments, consuming the energy of ATP formed during glycolysis, respiration, and photosynthesis to carry organelles, proteins, and metabolites including lipids and polysaccharides, to all parts in the cell^34,35^ (Fig. S14b). In this study, we unexpectedly observed that followed by Ψ_p_ loss, a transient increase in *V*_*Mito*_ (i.e., matrix volume) occurred contrastingly with the lowering of *V*_*o*_, Ψ_w,_ and Ψ_s_ (see Fig. 1), maintaining cytoplasmic streaming velocity in tomato trichome stalk cells subjected to the salt stress treatment (Fig. 1f). Furthermore, the relationship between *V*_*o*_ and the water potential gradient between the water source and the cells (i.e., Ψ_o_ − Ψ_w_) was shown to be a hysteresis (Fig. S15a). When the *V*_*Mito*_/*V*_*o*_ throughout swelling and shrinking was also plotted against the water potential gradient, an inverse hysteresis loop has been synchronously found (Fig. S15b). Hence, mitochondria moving in the cells likely perceive such a gradient to regulate their volume during the reversible salt stress response (see Fig. S14).

There is accumulating evidence that an increase in K^+^ and Ca^2+^ influx play a crucial role in mitochondrial swelling^36^. Taken together with our data and the previous reports on the mitochondrial osmotic and hydraulic regulations^37,38^, the reversible *V*_*Mito*_ changes could be attributed to the transient water inflow into the matrix through AQPs localized in IMM and permeability transition pores (PTPs) localized in the outer mitochondrial membrane (OMM) in mitochondria^39,40^, in accordance with the water potential gradient, presumably associated with enzymatic cascade reactions by the ion channels. Increases in IMM permeability might also be accompanied with the increase in Ca^2+^ influx^41^. The recent model reveals the dynamics of mitochondrial swelling caused by an increase in the colloid osmotic pressure and the IMM rigidity, together with the dynamics of the ionic/neutral species^42^. In this study, the exact role of these ionic species on the mitochondrial volume regulation was not determined. When *V*_*o*_ reduces, cytosol should be more concentrated, but with mitochondrial swelling by osmosis (see Fig. 1). When calculated the increased solute concentration based on the reduction in Ψ_s_ (*Δ*Ψ_s_ = 0.37 MPa in 1 h, see Fig. 1d) under the growing conditions (28 °C) according to the van’t Hoff relation, the extent of increases in mitochondrial solute concentration should be greater than 147 mM to ensure the water flow into the mitochondria. It has been also accepted that the opening of mitochondrial PTP triggered by Ca^2+^ might be potentiated by reactive oxygen species. The redox-related cellular parameters suggest that this potentiation might be ignorable at least in the reversible swelling observed in this study (see ASC/DHA and GSH/GSSG ratios in Fig. 2).

**Figure 5:**
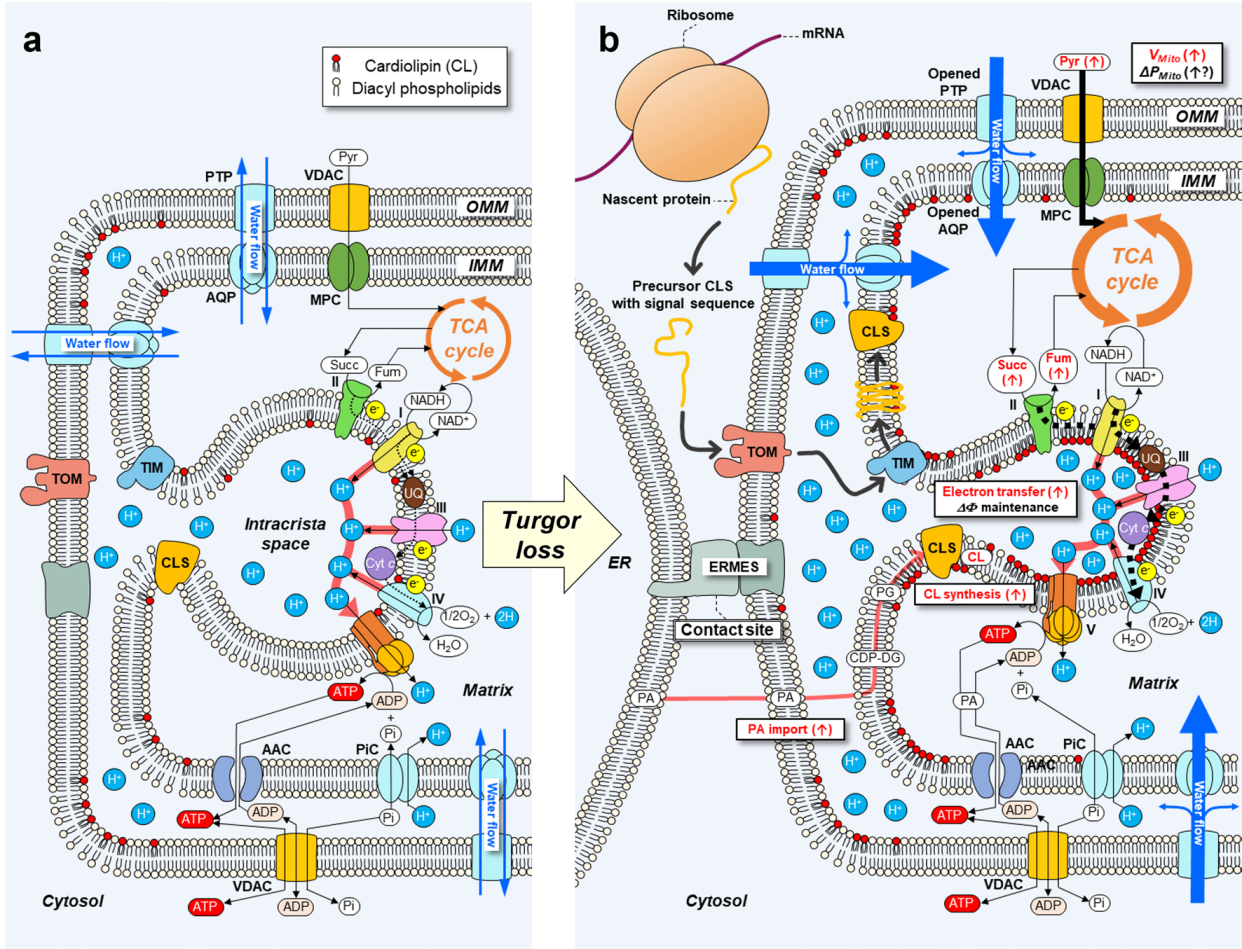
Putative model of mitochondrial volume regulation and electron transport chain associated with turgor-driven cardiolipin (CL) biosynthesis. Mitochondrial energy production^46^ under normal conditions (**a**) and salt stress conditions (**b**). Turgor loss may be transmitted to the nucleus and membranes as a hydraulic signal, which may upregulate nuclear cardiolipin synthase (CLS) gene expression^47^ as well as the opening of putative permeability transition pore (PTP)^39^ and aquaporins (AQPs)^38^ in each mitochondrial membrane (see text). These responses would lead to temporal expansion in mitochondrial volume (*V*_*Mito*_) and CL accumulation. In the cytosol, the newly translated precursor CLS proteins would be imported into inner mitochondrial membrane (IMM) through translocase of the outer membrane (TOM)^48^ and incorporated into IMM through translocase of the inner membrane (TIM)^48^. And, the three-dimensional structure of CLS would be properly formed in IMM to exhibit enzyme activity. At the endoplasmic reticulum (ER)-mitochondrial connection, phosphatidic acid (PA) may be imported from ER to IMM through outer mitochondrial membrane (OMM)^49^, and thereafter PA would be synthesized throughout several steps in phospholipid biosynthesis to form CL, resulting in an increase in the curvature of IMM^50^. The different dipole moments and dipole density both cause a net polarization^51^. The maintenance of mitochondrial membrane potential difference (*ΔΦ*) streamlines electron transfer and H^+^ transfer by respiratory chain complexes (see Fig. S16 for more details). As the result, the maintenance of H^+^ gradient contributes to ATP synthesis even under salt stress conditions, sustaining the cytoplasmic streaming velocity. VDAC; voltage-dependent anion channel; MPC: mitochondrial pyruvate carrier; TCA: tricarboxylic acid; Pyr: pyruvate; Succ: succinate; Fum: fumarate; NAD^+^: nicotinamide adenine dinucleotide; NADH: nicotinamide adenine dinucleotide hydrate; UQ: ubiquinone; Cyt-*c*: cytochrome *c*; ADP: adenosine 5’-diphosphate; PiC: phosphate carrier; AAC; mitochondrial ADP/ATP carrier; ERMES: ER-mitochondria encounter structure^52^; mRNA: messenger ribonucleic acid.

In addition to the close relationship between the ion influx and matrix swelling, it is proposed that increases in mitochondrial matrix volume would be closely associated with the changes in cristae morphology through the structural organization of CL^43^. High concentration of pyruvate detected at the cell level (Fig. 2) likely refers to an active pyruvate influx into the matrix through the mitochondrial pyruvate carrier (MPC), and greater accumulation of succinate and fumarate suggests the enhancement of TCA cycle (see Figs. 2 and 5). The synchronous increase in PG and CLs concentration (Figs. 2 and 4) would be explained by the active incorporation of newly synthesized CL into IMM through the upregulation of gene expression, resulting in its morphofunctional modification. The incorporated CLs would contribute to the IMM curvature formation and also play as an effective proton trap^6^, resulting in the enhancement of electron transfer system during oxidative phosphorylation, even though the cells were more concentrated and viscous at salt stress (Figs. 2, 5, and S16)^6^. These mitochondrial compositional and morphological changes are strikingly evident for sustainable energy production in response to the salt stress, consistent with the previous reports^33,44,45^.

In *Solanum* spp., cardiolipin synthase (CLS) genes are assumed to be located in the nucleus^53^. *ΔT*_*df*_ for CL accumulation was 0.92 h (Fig. 4), which would be long enough to enhance CL synthesis including protein translocation illustrated in Fig. 5. Considering the several lines of evidence on Ψ_p_-associated gene expression^13–17,54^, the most plausible explanation is that pressure difference might be perceived by membranes and the nuclear envelope, leading to the upregulation of CLS gene expression. Assuming that the circumference along a central vacuole corresponds to 670 μm based on the vacuolar dimension (298 μm in length × 37 μm in width), the cytoplasmic streaming velocity is 4.57 μm/s (Fig. 1f), and hence the time required for mitochondria travel around the periphery of the vacuole during cytoplasmic streaming (Figs. 1f and S14b–d) is 2.44 min per round. Each mitochondrion in motion should temporally have a contact site^49,52^ tethered with the endoplasmic reticulum (ER) membrane (Figs. 5 and S14b–d). Precursor CLS proteins with a signal sequence translated at ribosome would be targeted to mitochondria to be imported from ER into the mitochondria through the translocase of the outer membrane (TOM) embedded in OMM^47,48^, and thereafter, mature CLS proteins would be incorporated in IMM through the translocase of the inner membrane (TIM), and CLS proteins are likely to maintain the activity (Fig. 5b). Thus, it appears that the CL accumulation occurred at the completion of osmotic adjustment (see *ΔT*_*df*_ for cell Ψ_s_ = 0.47 h in Fig. 4). If changes in cellular redox states are significant in CL synthesis, misfolded proteins would be produced. However, this is not the case because of essentially no influence in cellular redox states throughout treatment (Fig. 2), as addressed above. Although one would expect that CL might be remodeled, this seems unlikely when considering that the turnover of CL that was much slower than other phospholipids^55^. Taken together with the previous reports^13–17,47^, Ψ_p_ loss might be responsible for temporally upregulating the gene expression of CL that maintains mitochondrial oxidative phosphorylation, as the data show, again with no reduction in cytoplasmic streaming under the stress conditions (see Fig. 1f).

The analytical method, termed picoPPESI-MS, utilized here (Fig. S2) is a rapid and powerful analysis based on the combination of Orbitrap mass spectrometer and CPP that is a device long used for determining cellular water status in plants^23,30^. By adopting an ESI–MS emitter electrode embedded in the microfabricated capillary tip used for CPP filled with silicone + ionic solution^25^, it is noteworthy that the system is capable of determining numerous cell metabolites after knowing the water status of the cells, where the tip was impaled. The robustness of picoPPESI-MS has been demonstrated in the snap-shot analysis in various plant cells^25,27,28,31,56^, but not for time-course analysis. Retaining the function of a CPP, the method can be applied to the cells attached to the plants to avoid possible artifacts that might occur at tissue preparation including cutting. Technically, it is true that the tip impalement gives damage to the cells during the CPP operation; however, the damage by CPP is likely negligible, as Shackel and his coworkers showed Ψ_p_ maintenance (91–96%) after puncture wounding due to the tip impalement in *Tradescantia* leaf epidermal cells^57^.

In addition, picoPPESI-MS is the only available technique so far that allows to investigate the kinetics of changes in both water status and metabolisms at the cell level in growing plants, along with the synchronous determination of *V*_*o*_ and velocity of cytoplasmic streaming. The tip removal at cell sap extraction had been completed within 0.5 s in this study, much shorter than half time (*T*_1/2_) in most plant cells including the cells used here (see Fig. S17). For organophosphate metabolites (Glc6P, Fru6P, Glc1P, and ATP), some minor fragmentation has been observed in the picoPPESI process (see Figs. S4, S5-1, S5-2, and S7). Therefore, the system has the ability to detect a transient increase in ATP content (see at 0.5 h in Fig. 2 ATP) even though ATP has a rapid turnover rate (i.e., *T*_1/2_ = 0.28 s)^58^. The combined analysis of picoPPESI-MS and TEM has strongly suggested that mitochondria serve as the major organelle in the cellular fluids collected in the microcapillary tip even with the tissue fixation process (see Fig. S9 and Table S2).

In single-cell metabolomics, it has been challenging to simultaneously determine CL-like phospholipids and other numerous metabolites contained in the picolitre solution without any pretreatment in the growing plants, as > 300 metabolite-related ions formed in the cloud phase have been detected during the ESI event in one single shot (see Table S1) after collecting the relatively small portion of cellular fluids (between 11.4 and 15.4% of *V*_*o*_). By utilizing the ‘volume control using a *transparent* microcapillary tip’ in CPP (i.e., picoPPESI-MS), for the first time, the concentration of CL-like phospholipids has been accurately evaluated at the single-cell level (see Fig. 1h and i) and demonstrated in the living cells that CL would be responsible for the structural IMM modification, confirming the model proposed^50^. This was achieved by coupling with the volume determination of the picolitre cellular fluids discharged in the probe tip by identifying/controlling the position of the meniscus formed between oil (silicones) and cellular fluids under the digital microscope during CPP operation. In this study, ca. 2 μm i.d. probe tip was pointed exactly towards the Orbitrap mass spectrometer inlet (0.58 mm i.d.) with the approximately 4 mm distance, and immediately ESI ionization was initiated by applying a high voltage into the capillary internal ionization electrode^25^. There was a linear correlation between signal intensity and the concentration for each phosphate metabolite including ATP in the standard solutions (see Fig. S4), which was obtained by fixing the sap volume to be equivalent, and was evaluated (Fig. S2). Some suppression phenomena might have occurred on each metabolite in the ESI process in the real samples used; however, considering the distance between the capillary tip i.d. and the mass spectrometer inlet, and their alignment, it is plausible to consider that this effect is minimum, and in a reproducible way, the majority of ions generated are introduced into the Orbitrap analyzer (Figs. 2 and S2 and Table S1).

Over two centuries, cytoplasmic streaming has been long recognized as an essential phenomenon in plants, from algae to angiosperms^34^. Trichomes have been used as a model system to study the cellular dynamics of various environmental stresses^59^. In this system, the interaction between cytoplasmic streaming, cell water relations, and metabolisms under other stress conditions (e.g., drought) remains to be established in future work. Because of the lack of a suitable direct cellular analysis, little was known about the exact cellular mechanics on Ψ_p_-induced gene expression in the past. In this study, Ψ_p_-associated CL biosynthesis has been identified based on the kinetics of related cellular parameters (Figs. 4 and 5). And, it has been shown for the first time that there is a hysteresis in mitochondrial volume versus the water potential gradient during reversible salt stress response (Fig. S15). Currently, the picoPPESI-MS system is confined to cell water status measurement and single-cell metabolomics. Further improvement for incorporating the function of proteomics and transcriptomics into the current system will be promising. In the view of cell water relations, conducting such a single-cell multi-omics analysis will further extend our knowledge of the cellular dynamics in living plants in response to changing environments.

In conclusion, we used trichome cells in the intact tomato plants as a model system to examine the cellular dynamics in response to salt stress from the viewpoint of plant water relations. Performing a time-course picoPPESI-MS analysis combined with TEM allowed to determine the kinetics of physiological parameters and metabolisms at the single-cell level. Results indicate that Ψ_p_ loss preceded *V*_*o*_ reduction accompanied with a temporal expansion in *V*_*Mito*_ at osmotic adjustment, which leads to the IMM morphological changes throughout the CL accumulation, resulting in keeping the cytoplasmic streaming velocity unchanged under salt stress conditions. Hence, it is concluded that changes in Ψ_p_ are responsible for optimizing the mitochondrial energy production in response to given stress conditions in plants.

## Methods

### Plant materials

A model cultivar for tomato (*Solanum lycopersicum* L. cv. Micro-Tom WT (Strain ID: TOMJPF00001)) seeds were provided by the National BioResource Project (NBRP) tomato program at the University of Tsukuba, Japan under a standard material transfer agreement and an Institutional NBRP Policy for ABS of plant genetic material following international agreement signed by Japan. The seeds were sown on a humidified growth medium consisting mainly of peat moss (Sakata seed Corp., Kanagawa, Japan) and incubated in a growth chamber (MLR-352H, Panasonic Corp., Osaka, Japan) in Ehime University. In the chamber, air temperature, relative humidity, photosynthetic photon flux density, and photoperiod were set at 28 °C, 60%, 115 μmol m^−2^ s^−1^, and 16 h, respectively. Two-week-old seedlings were then grown hydroponically in the chamber for two weeks. For the hydroponic solution, fourfold dilution of the modified Hoagland solution ((in mM) 7.50 NO_3_^−^, 2.50 K^+^, 2.50 Ca^2+^, 0.50 PO_4_^3-^, 0.50 SO_4_^2−^, 0.50 Mg^2+^, and (in μM) 2.50 Fe^2+^, 0.13 Mn^2+^, 0.75 BO_3_^−^, 0.25 MnO_4_^2−^, 0.10 Zn^2+^, 0.05 Cu^2+^) was used as control (the media water potential, Ψ_o_^cont^ = − 0.05 MPa). By exchanging the control solution to 1X Hoagland solution treatment (the media water potential, Ψ_o_^stress^ = − 0.14 MPa), plants were subjected to salt stress conditions (i.e., treatment difference in media water potential, *Δ*Ψ_o_ = 0.09 MPa). Ψ_w_ of each media solution was determined by isopiestic psychrometry^60^, and the pH of each media solution was adjusted to 6.0 ± 0.5 (*n* = 11).

### In situ determination of cell water status

Simply, tomato plants were placed on a vibration-free table, and the lowest position of single stalk cells of type II trichome on the adaxial surface of the sixth leaf counting from the bottom of plants were viewed under a digital microscope (KH-8700, HIROX Co. Ltd., Tokyo, Japan) (Fig. S2). Quartz microcapillary of 1.0 mm in outer diameter and 0.7 mm in inner diameter was used, and the microcapillary tip was reproducibly fabricated by using a laser micropipette puller (P-2000, Sutter Instrument Co., CA). The set-up of a CPP was similar to the previous work^25^. The microcapillary tip filled with 0.01% (v/v) ionic liquid/silicone oil mixture was broken open to be ca. 2 μm i.d. by gently crashing onto the edge of styrofoam. Prior to the tip insertion, oil pressure in the microcapillary was set at slightly lower than the putative Ψ_p_ value in the target cells, and the traveling vesicles in focus were videotaped for > 5 s, so that later the vesicles could be randomly selected for determining their moving rate as the cytoplasmic streaming velocity (Movie S1). And thereafter, the tip was inserted into the cells. Once the tip was inserted into the target cells, the boundary (i.e., meniscus) formed between the oil and aqueous fluids discharged from the cells was formed, which was identified through the transparent microcapillary. By increasing the oil pressure by the means of CPP, the meniscus could be moved back to the correct position to determine Ψ_p_ in the impaled cells^23,60^. This pressure manipulation using a transparent microcapillary tip is quite important in order to (i) minimize the potential artifacts from hydraulic disturbances in the cells that might occur at the tip impalement and (ii) determine the volume of cellular fluids collected in the microcapillary tip, as described below. After the determination of Ψ_p_, oil pressure was reduced to subzero pressure, and instantly the microcapillary tip was removed from the cells.

An aliquot of the cellular fluids discharged into the tip was then quickly transferred onto a cryo-osmometer silver plate filled with high viscous microscopic oil (Type B Cargille Immersion Oil) to determine the cell Ψ_s_ by using a nanoliter freezing point osmometer (Clifton Technical Physics, Hartford, NY)^61,62^. The cell Ψ_w_ was calculated by summing Ψ_p_ and Ψ_s_^63^. Under the microscope, the entire trichome was videotaped to estimate the *V*_*o*_. All these parameters described above were measured at 0, 0.5, 1, 3, 6, and 12 h after the salt stress treatment. In some cases, wall elastic modulus (*ε*) and cell hydraulic conductivity (*L*_*p*_) were measured with a CPP in control cells (*T* = 0 h). By applying < 0.1 MPa of pressure pulses, *ε* was determined by *ε* = *V·ΔP/ΔV* after the Ψ_p_ measurement according to the previous studies^29,64^. The *L*_*p*_ is given by *L*_*p*_ = ln(2)*V*/(*AT*_*1/2*_(*ε* + *π*))^29^, where *A* is the cell surface area, and *π* is the cell osmotic pressure (i.e., − Ψ_s_) determined with the nanoliter osmometer. Performing endosmotic and exosmotic pressure relaxations with approximately ± 0.05 MPa of pressure to avoid hydraulic disturbance^12^, *T*_*1/2*_ was determined for the *L*_*p*_ measurement^29,30^.

### In situ cell metabolomics

At 0, 0.5, 1, 3, and 6 h after the salt stress treatment, immediately after the Ψ_p_ assay and cytoplasmic streaming velocity determination in about half of the cases, ca. 200 pL of cell sap was collected by depressurizing oil pressure in the microcapillary, and the image was taken. The preliminary experiment showed that the volume of cellular fluids corresponded to approximately 200 pL if the distance between the tip and meniscus location in the capillary was 500 μm in all the microcapillary tips reproducibly produced. The tip was rotated to be pointed toward the inlet of the Orbitrap mass spectrometer (Exactive Plus, Thermo Fisher Scientific Inc., MA, the US) (see Fig. S2) and electrified through the internal electrode with − 4 kV using a high voltage generator (AKTB-05k1PN/S, Touwa Keisoku Corp., Tokyo, Japan), and consequently, the metabolite ions by the ESI process are generated and cell metabolomics was carried out by using picoPPESI-MS^25^. The mass spectra were acquired in negative ion mode with the instrumental settings of 50 ms as maximum injection time, inlet ion transfer tube temperature of 250 °C, resolution of 35,000, and automatic gain control (AGC) value of 1 × 10^6^. When Ψ_p_ assay was succeeded without tip plugging, the entire process of picoPPESI-MS analysis on the cells was completed within 3 min.

In addition, a close inspection was conducted to identify the type and variation of organelles in the fluids collected in the microcapillary tip. Under the microscope, cellular fluid was loaded onto the formvar film placed on the TEM grid and stained with lead citrate, and viewed with a TEM (JEM-1011, JEOL Ltd., Tokyo, Japan).

Tandem MS experiments were conducted on phosphoric acid compounds (NaH_2_PO_4_ and Na_2_HPO_4_) and the stalk cell sap under the same experimental conditions. Collision-induced dissociation tandem MS analysis (MS/MS analysis) in negative ion mode was performed using an Orbitrap mass spectrometer (Orbitrap Elite, Thermo Fisher Scientific Inc., MA, the US) coupled with the picoPPESI system. The MS/MS scan spectra were acquired with the instrumental settings of 100 ms as maximum injection time, inlet ion transfer tube temperature of 275 °C, resolution of 60,000, and AGC value of 5 × 10^4^.

The standard chemicals for MS analyses, NaH_2_PO_4_ and Na_2_HPO_4,_ were purchased from Wako Pure Chemical Industries, Ltd. (Osaka, Japan). Glucose 6-phosphate (Glc6P), glucose 1-phosphate (Glc1P), fructose 6-phosphate (Fru6P), and ATP were purchased from Sigma-Aldrich Japan (Tokyo, Japan). Water, liquid chromatography-mass spectrometry (LC/MS) grade was purchased from Thermo Fisher Scientific Inc. (MA, the US). For picoPPESI-MS operation, the ionic liquid, trihexyl (tetradecyl) phosphonium bis trifluoromethanesulfonyl amide (Cyphos IL109 Strem Chemicals Inc., MA, the US), was suspended in phenylmethyl silicone oil (Wacker silicone fluid AS4, Munich, Germany) at a concentration of 0.01% (v/v) to enhance electric conductivity of the silicone oil.

Exact monoisotopic *m/z* values for all the peaks on the mass spectra acquired were extracted using Xcalibur Qual Browser (Thermo Fisher Scientific Inc., MA, the US). Metabolites were identified from the theoretical masses of candidate metabolites in METLIN database (http://metlin.scripps.edu/index.php), allowing differences of < 5 ppm, and the limit of detection was determined by the signal intensity of the substance that reaches at least 3 times the signal noise of the baseline. The ratio of ASC/DHA and GSH/GSSG was determined according to the previous work^56^. Linear regression was performed in SigmaPlot 13.0 (Systat Software Inc., CA, the US, https://systatsoftware.com/sigmaplot/). A heatmap was generated with Python 3.9.1 (https://www.python.org/) using Matplotlib and Seaborn.

### Microscopic observations

At 0, 0.5, 1, 3, 6, and 12 h after salt stress treatment, the same leaf (see above) tissues were sampled for the following microscopic observations in the stalk cells of type II trichome. In the preliminary experiment, Ψ_s_ of the stalk cells in each timing was determined by using the nanoliter osmometer. The leaf tissues containing trichomes were fixed for 2 h at 25 °C with 3% (v/v) glutaraldehyde with 50 mM sodium phosphate (pH 7.2) containing sucrose to adjust each trichome and the fixation solution, to be similar to the observed tissue Ψ_s_, typically within ± 0.10 MPa^65^. And thereafter, the tissues were washed in 100 mM sodium phosphate (pH 7.2) and fixed with 2% (v/v) osmium acid and 50 mM sodium phosphate (pH 7.2). The fixed tissues were dehydrated through an ethanol series and embedded in LR White resin (London Resin, Hampshire, the UK) for one-day polymerizing at 65 °C. Ultra-thin Sections (100–120 nm) for electron microscopy were stained with lead citrate and observed with the above-mentioned TEM.

For the organelle image analysis, the vacuoles, nucleus, mitochondria, plastid, and peroxisome in the same stalk cells were observed microscopically. For mitochondria, *V*_*Mito*_, the number per cell, and *V*_*Mito*_/*V*_*o*_ were measured using an image processing software, ImageJ version 1.53 k (US National Institutes of Health, Bethesda, MD, the US, https://imagej.nih.gov/ij/). For the internal structure of mitochondria, the intracrista space area, the number of cristae, intracrista area per mitochondrion, *τ*, and *θ* were measured by using ImageJ version 1.53 k. *τ* was simply determined as the arc-chord ratio (see Fig. 3c and d): the ratio of the length of the curve to the distance between its ends, corresponding to each of crista apex. *θ* was determined as the average angle measured at the beginning and end points of the long diameter of intracrista space^33^. With the time-course changes in *V*_*o*_ and *V*_*Mito*_/*V*_*o*_, the relationship of *V*_*o*_ and *V*_*Mito*_/*V*_*o*_ versus the water potential gradient was examined individually. Violin plots were created with Python 3.9.1 using Matplotlib and Seaborn. Linear regression and nonlinear curve fitting by Hill equation were performed in SigmaPlot 13.0.

### Tissue water status and transpiration measurements

Leaf tissue segments from the same leaf (see above) were gently collected using a 4.0 mm i.d. cork borer at 0, 6, and 12 h after treatment. All subsequent tissue manipulations were performed under the humid chamber to minimize water loss from the tissue after excision^60^. The tissue water status was then determined with the isopiestic psychrometer^60,66^. After Ψ_w_ was measured, tissue Ψ_s_ was determined in the same tissue immediately after freezing at − 80 °C and thawing at 25 °C. Ψ_p_ was calculated by subtracting Ψ_s_ from Ψ_w_^60^. At − 0.5, 0, 0.5, 1, 3, 6, and 12 h after treatment, water loss rate due to the transpiration was also determined from the weight changes in the whole plant per time in each treatment by using an electronic balance (LC-1200S, Sartorius AG, Göttingen, Germany). Images of all the leaves collected at 12 h were acquired with a scanner and then binarized by using ImageJ version 1.53 k to calculate the total leaf area, and the transpiration rate (unit: g m^−2^ h^−1^) was determined at each timing.

### Statistical analysis

Analysis of variance with either Tukey–Kramer test or *t*-test was conducted for testing the significance of groups in water status measurement, metabolomics, and microscopic analysis. All statistical analyses were performed in R version 4.0.2 (https://www.r-project.org/).

### Plant ethics statement

All the experiments on plants, including the water status measurement and metabolomics conducted in this study were strictly performed in accordance with relevant guidelines, regulations, and legislation.

## Supplementary Information

Supplementary Information 1.

Supplementary Video 1.

Supplementary Information 2.

## Data Availability

The datasets used and/or analysed during the current study are available from the corresponding author on reasonable request.
