# Supplementary material for: Dynamics and stabilization mechanism of mitochondrial cristae morphofunction associated with turgor-driven cardiolipin biosynthesis under salt stress conditions
Source: Sci Rep. 2022 Jul 1;12:9727. doi: 10.1038/s41598-022-14164-3 (PMC9249792; doi:10.1038/s41598-022-14164-3)
Supplement: Supplementary file 3 — Supplementary Information 2. [file 41598_2022_14164_MOESM3_ESM.pdf]

Supporting Information for *Scientific Reports*

Dynamics and stabilization mechanism of mitochondrial cristae morphofunction associated with turgor-driven cardiolipin biosynthesis under salt stress conditions

Keisuke Nakata[1], Yuto Hatakeyama[2], Rosa Erra-Balsells[3], Hiroshi Nonami[2], and Hiroshi Wada[1,2*]

[1]The United Graduate School of Agricultural Science, Ehime University, Matsuyama, Ehime, Japan; [2]Graduate School of Agriculture, Ehime University, Matsuyama, Ehime, Japan; [3]Department of Organic Chemistry and CIHIDECAR-CONICET, University of Buenos Aires, Buenos Aires, Argentina

*Corresponding author (hwada@agr.ehime-u.ac.jp)

This PDF file includes:

Figures S1 to S17

Other supplementary materials for this manuscript include the following:

Tables S1 to S2
Movies S1

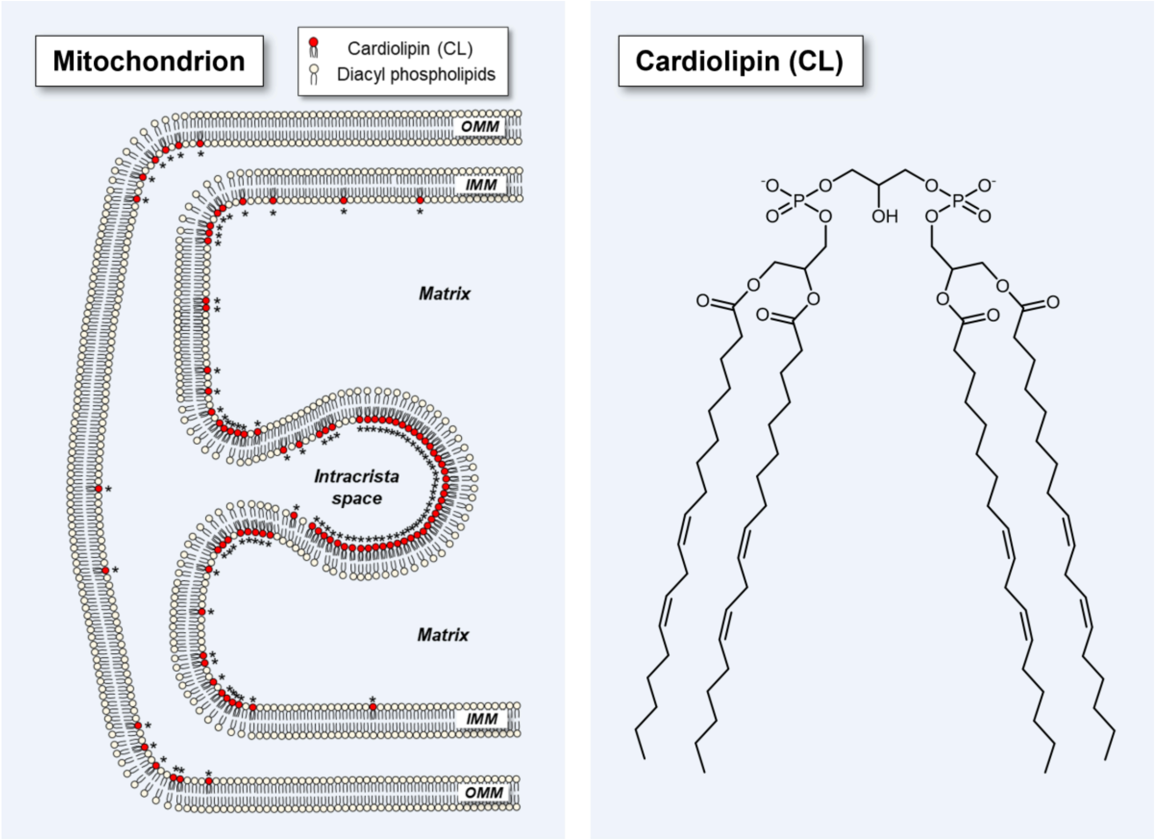

Figure. S1. Chemical structure of putative cardiolipin (CL) in the inner mitochondrial membrane (IMM). CLs are composed of three glycerol units and have four acyl side chains. CLs specifically exist in the inner mitochondrial membrane, where they regulate the morphology of the inner membrane, thereby enabling efficient energy metabolism. OMM: outer mitochondrial membrane. A chemical structure was drawn using ChemDraw 20.1.1 (PerkinElmer, Inc., MA, the US, https://perkinelmerinformatics.com/).

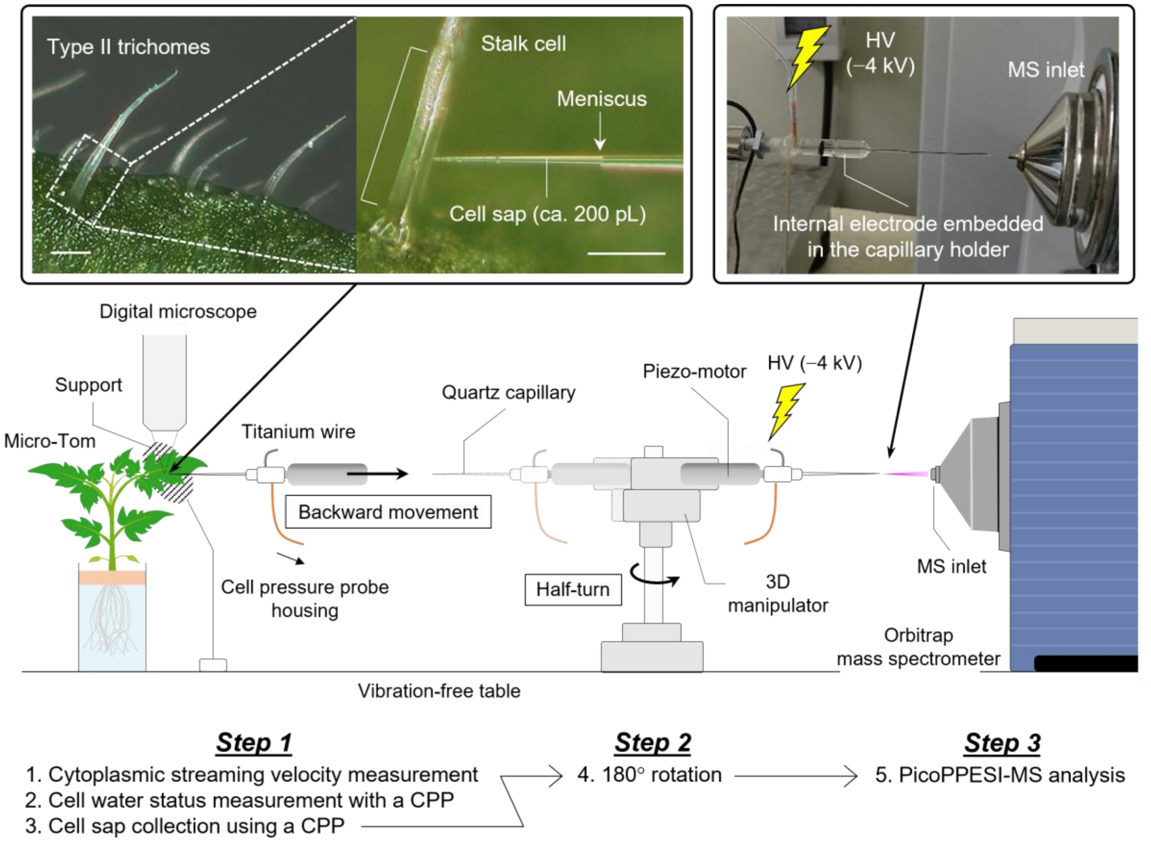

Figure. S2. A scheme of the experimental set-up used for in situ single-cell metabolomics termed picoliter pressure-probe electrospray-ionization mass spectrometry (picoPPESI-MS). Bars = 250 μm.

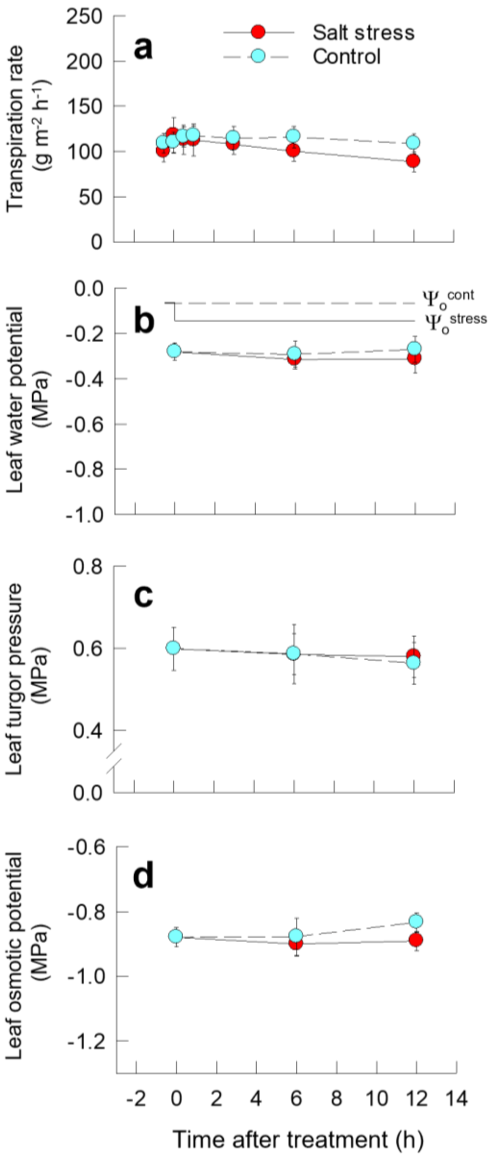

Figure. S3. Time-course of changes in transpiration rate (**a**), water potential (**b**), turgor pressure (**c**), and osmotic potential (**d**) of the leaf tissues (see Methods) under the salt stress conditions. Cyan circles indicate the control in the solution of $\Psi_o^{cont} = -0.05$ MPa; red circles indicate salt-stressed plants in the solution of $\Psi_o^{stress} = -0.14$ MPa. Data are means±SE for 4-8 plants in each treatment. Line graphs were created with Sigmaplot 13.0.

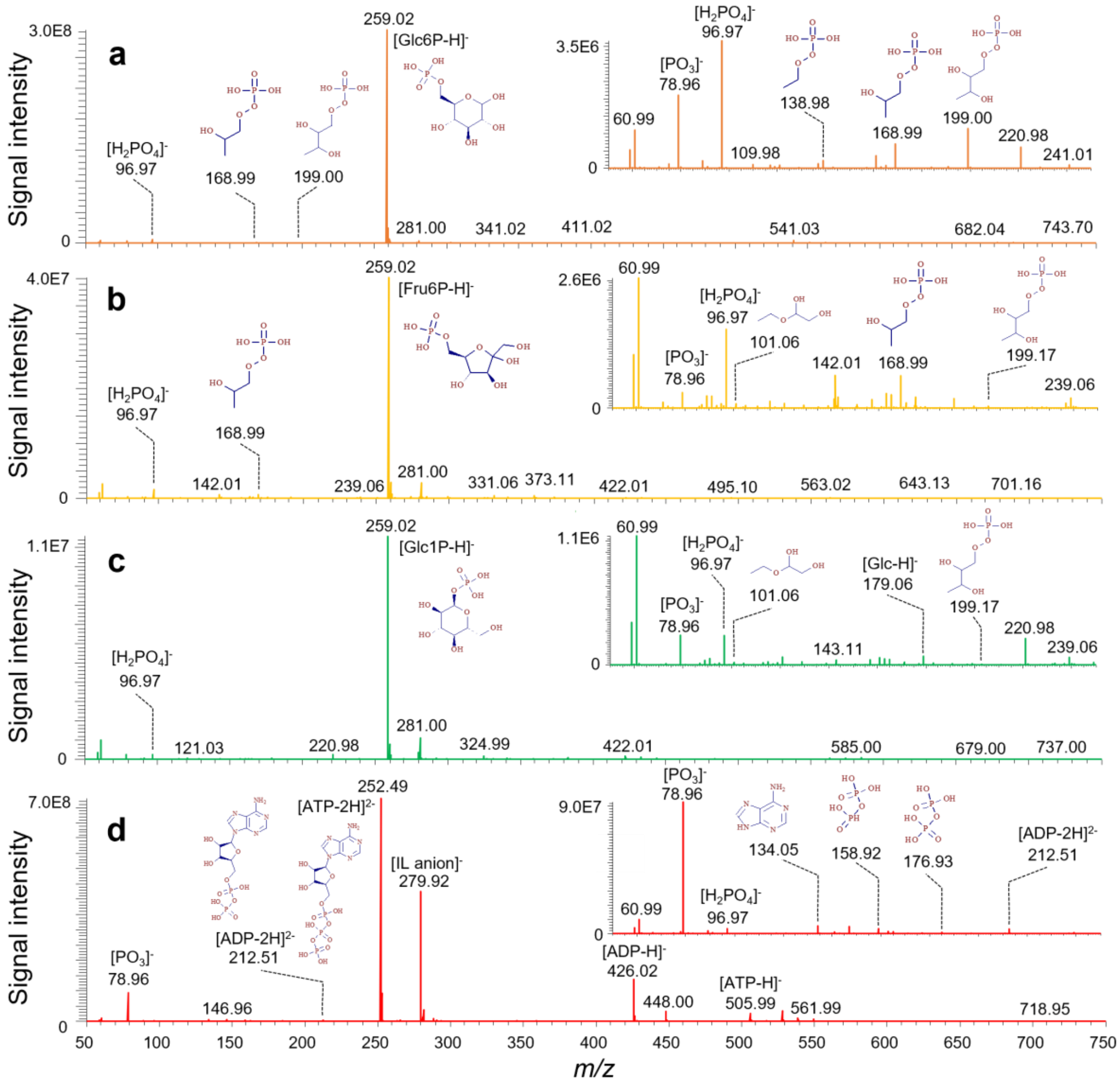

Figure. S4. PicoPPESI mass spectra of 1.0 mM standard solution of glucose 6-phosphate (Glc6P) (**a**), fructose 6-phosphate (Fru6P) (**b**), glucose 1-phosphate (Glc1P) (**c**), and adenosine 5'-triphosphate (ATP) (**d**) standard solution. Negative ion mode; solvent: water, LC/MS grade (Thermo Scientific); voltage: −4 kV; injection sample volume: ca. 200 pL. The chemical structures were drawn using Mass Frontier 7.0 (Thermo Fisher Scientific Inc., MA, the US).

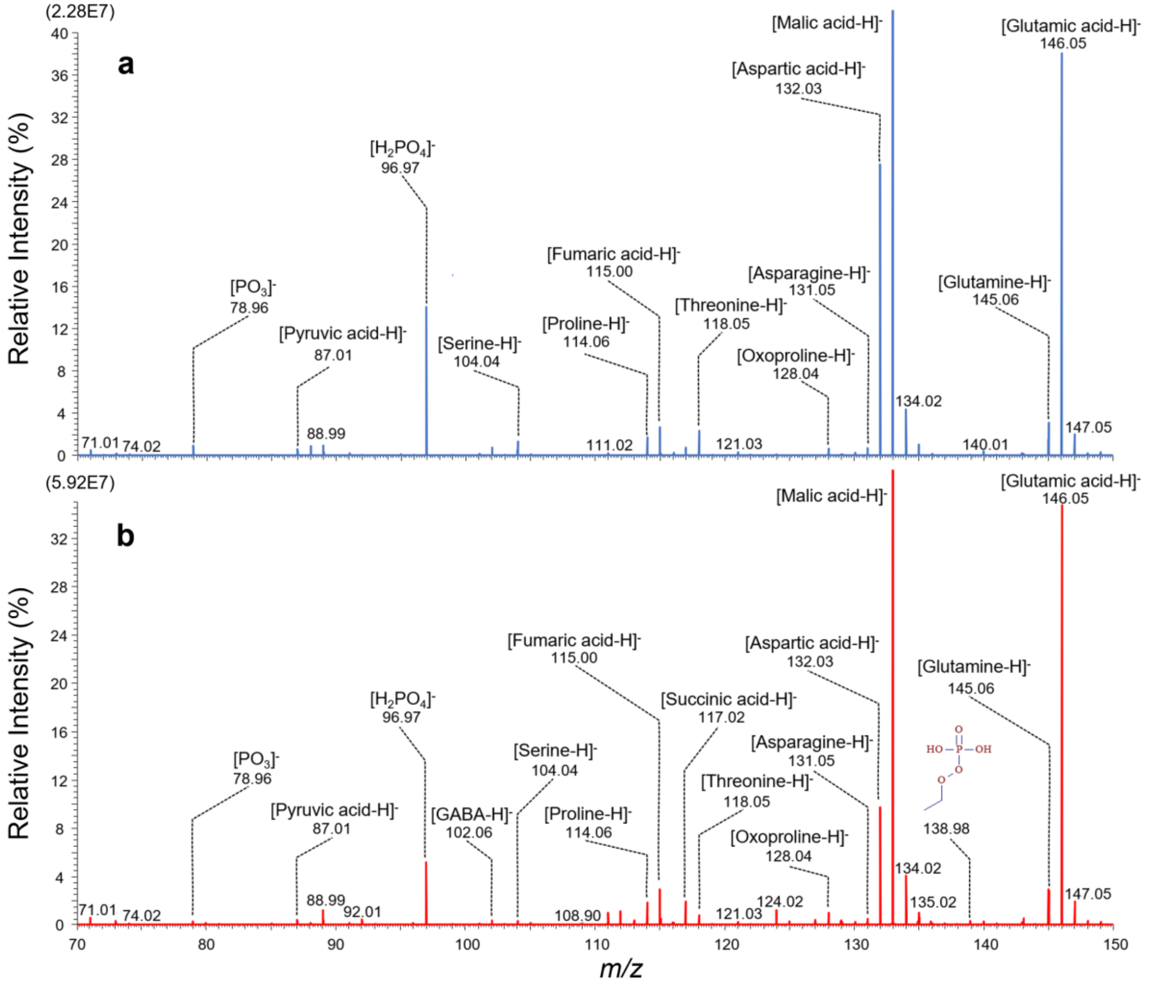

Figure. S5-1. The range of *m/z* 70-150 in picoPPESI negative ion mode mass spectra obtained from the stalk cells (see Methods) at 3 h in the control (**a**) and after the salt stress treatment (**b**). Voltage: −4 kV; injection sample volume: ca. 200 pL. Data represent repeated experiments with 8–32 stalk cells from 3–6 plants in each treatment. The full mass spectra are shown in Fig. 1. GABA: γ-aminobutyric acid. A chemical structure was drawn using Mass Frontier 7.0

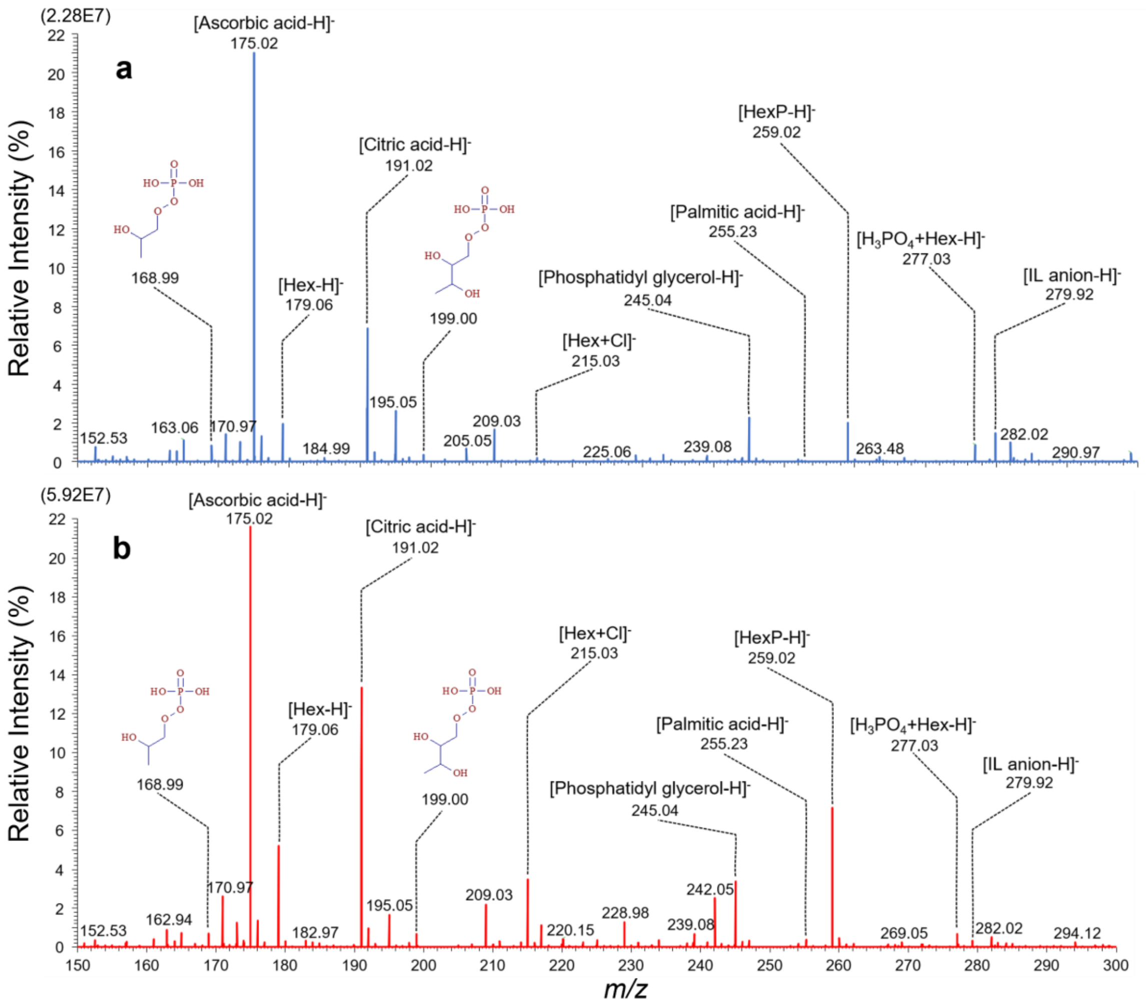

Figure. S5-2. The range of *m/z* 150-300 in picoPPESI negative ion mode mass spectra obtained from the stalk cells (see Methods) at 3 h in the control (**a**) and after the salt stress treatment (**b**). Voltage: −4 kV; injection sample volume: ca. 200 pL. Data represent repeated experiments with 8–32 stalk cells from 3–6 plants in each treatment. The full mass spectra are shown in Fig. 1. Hex: hexose; HexP: hexose phosphate; IL: ionic liquid. The chemical structures were drawn using Mass Frontier 7.0.

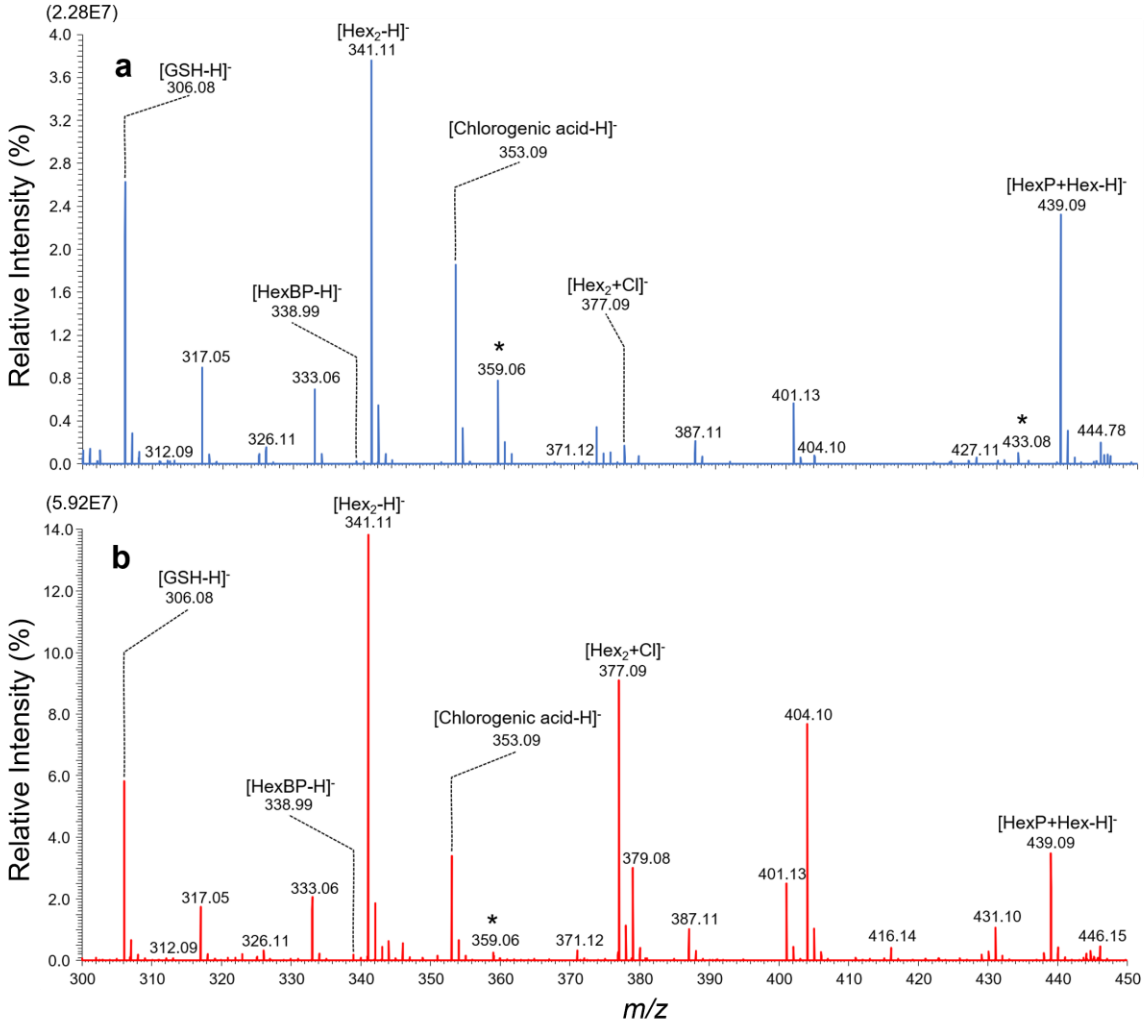

Figure. S5-3. The range of *m/z* 300-450 in picoPPESI negative ion mode mass spectra obtained from the stalk cells (see Methods) at 3 h in the control (**a**) and after the salt stress treatment (**b**). Voltage: −4 kV; injection sample volume: ca. 200 pL. Data represent repeated experiments with 8–32 stalk cells from 3–6 plants in each treatment. Asterisk markings indicate assigned background peaks from silicone oil+ionic solution filling into the pressure-probe capillary. The full mass spectra are shown in Fig. 1. GSH: glutathione; Hex: hexose bisphosphate; HexP: hexose phosphate.

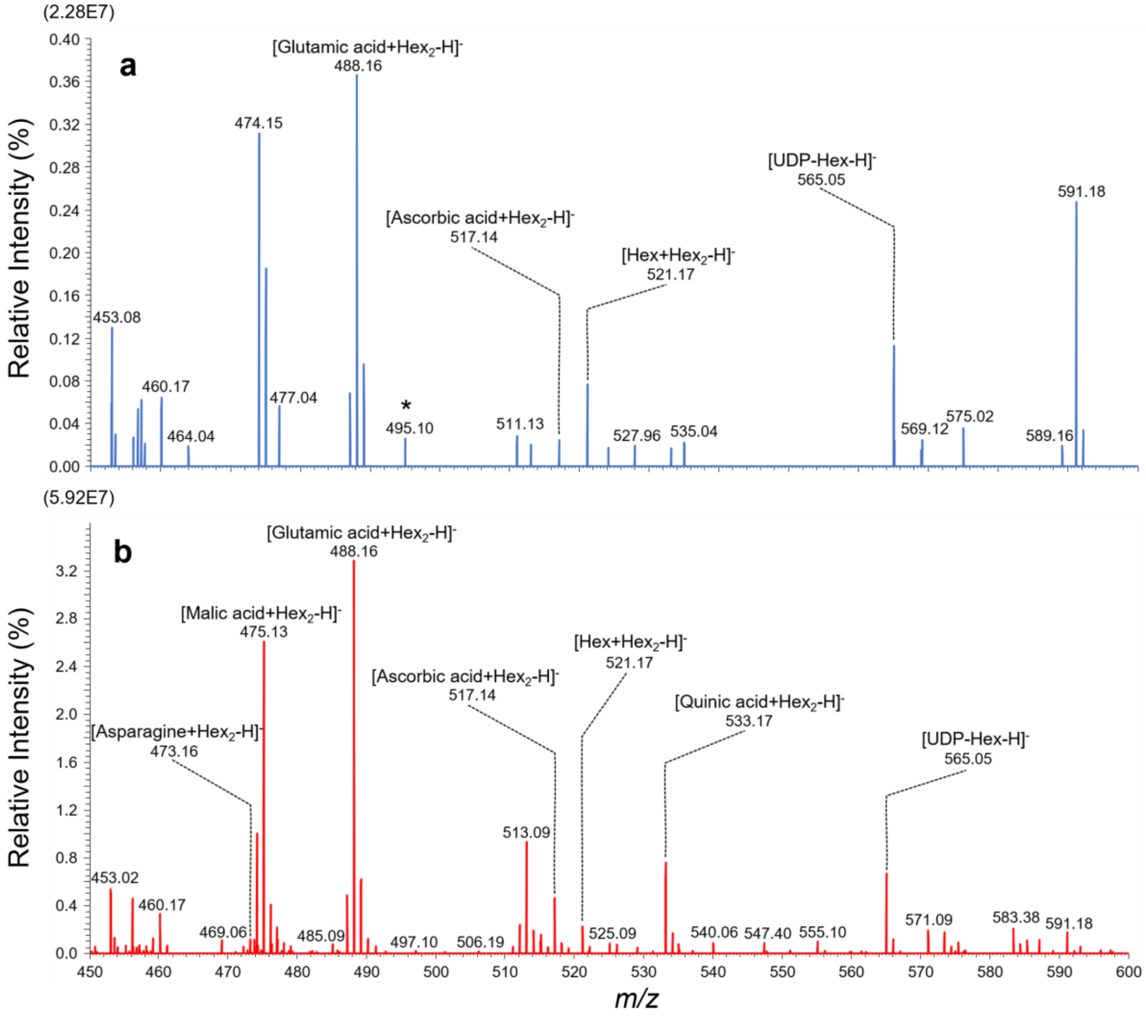

Figure. S5-4. The range of *m/z* 450-600 in picoPPESI negative ion mode mass spectra obtained from the stalk cells (see Methods) at 3 h in the control (**a**) and after the salt stress treatment (**b**). Voltage: −4 kV; injection sample volume: ca. 200 pL. Data represent repeated experiments with 8–32 stalk cells from 3–6 plants in each treatment. Asterisk markings indicate assigned background peaks from silicone oil+ionic solution filling into the pressure-probe capillary. The full mass spectra are shown in Fig. 1. Hex: hexose; UDP: uridine 5’-diphosphate.

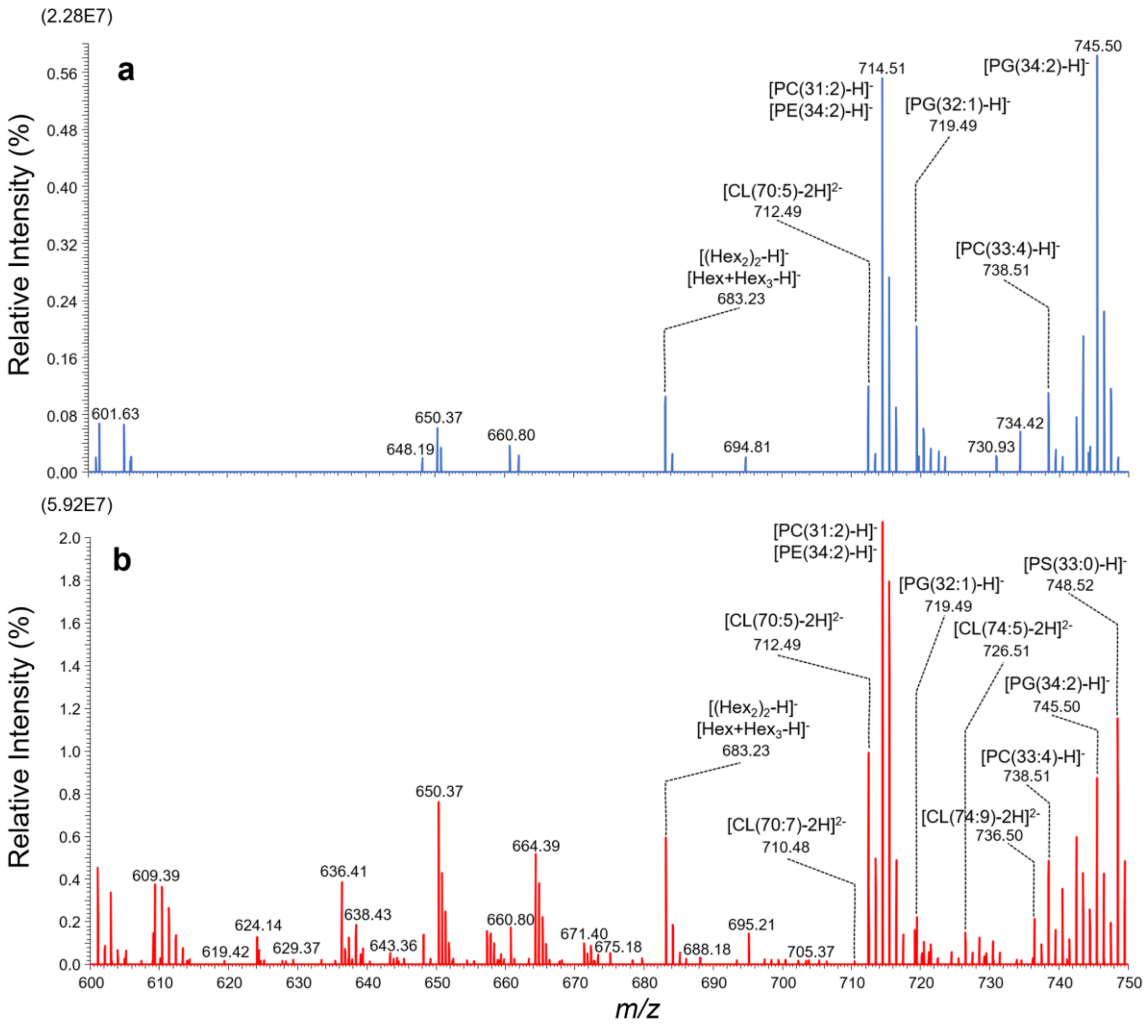

Figure. S5-5. The range of *m/z* 600-750 in picoPPESI negative ion mode mass spectra obtained from the stalk cells (see Methods) at 3 h in the control (**a**) and after the salt stress treatment (**b**). Voltage: −4 kV; injection sample volume: ca. 200 pL. Data represent repeated experiments with 8–32 stalk cells from 3–6 plants in each treatment. The full mass spectra are shown in Fig. 1. Hex: hexose; CL: cardiolipin; PC: phosphatidylcholine; PE: phosphatidylethanolamine; PG: phosphatidylglycerol; PS: phosphatidylserine.

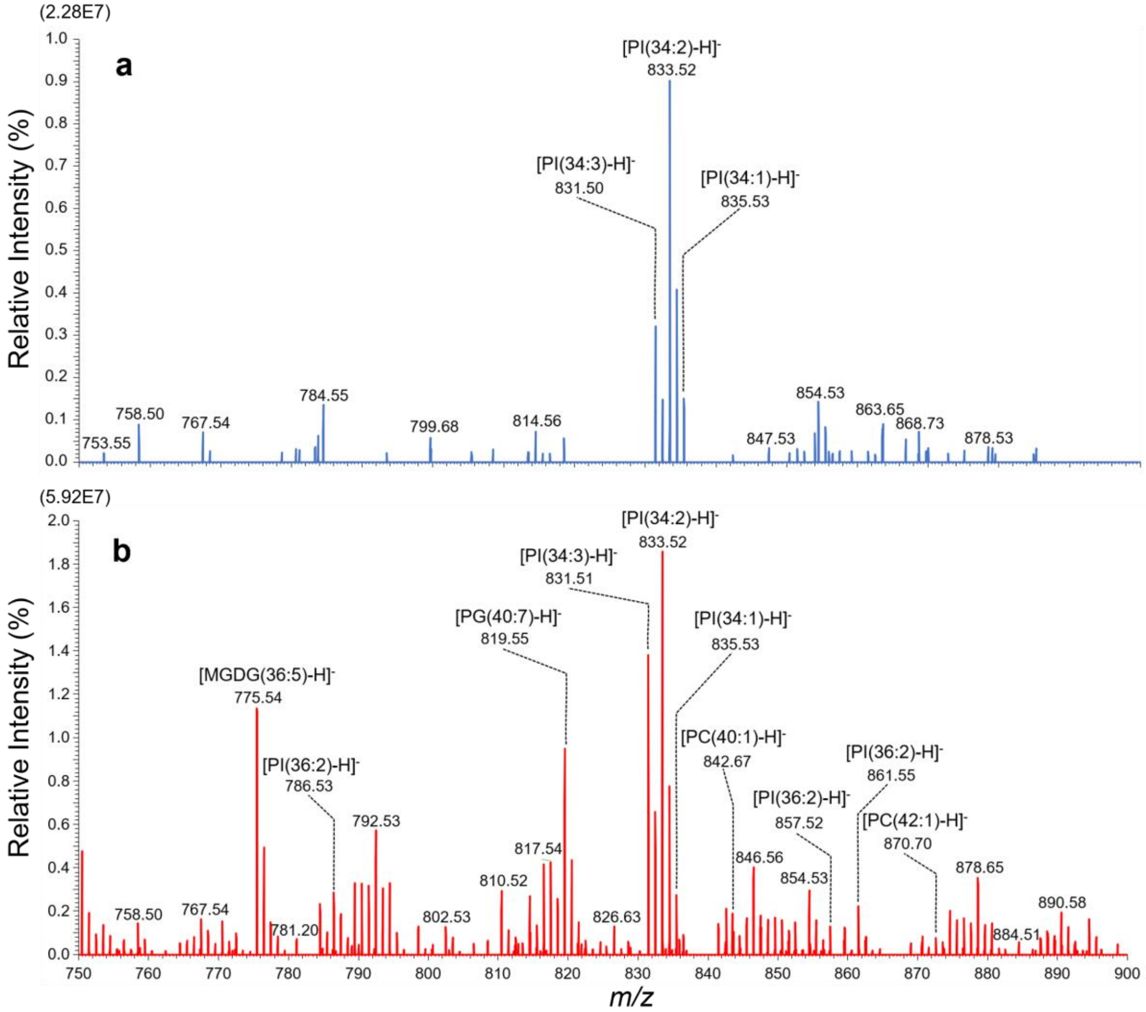

Figure. S5-6. The range of *m/z* 750-900 in picoPPESI negative ion mode mass spectra obtained from the stalk cells (see Methods) at 3 h in the control (**a**) and after the salt stress treatment (**b**). Voltage: −4 kV; injection sample volume: ca. 200 pL. Data represent repeated experiments with 8–32 stalk cells from 3–6 plants in each treatment. The full mass spectra are shown in Fig. 1. MGDG: monogalactosyl diacylglycerol; PI: phosphatidylinositol; PG: phosphatidylglycerol; PC: phosphatidylcholine.

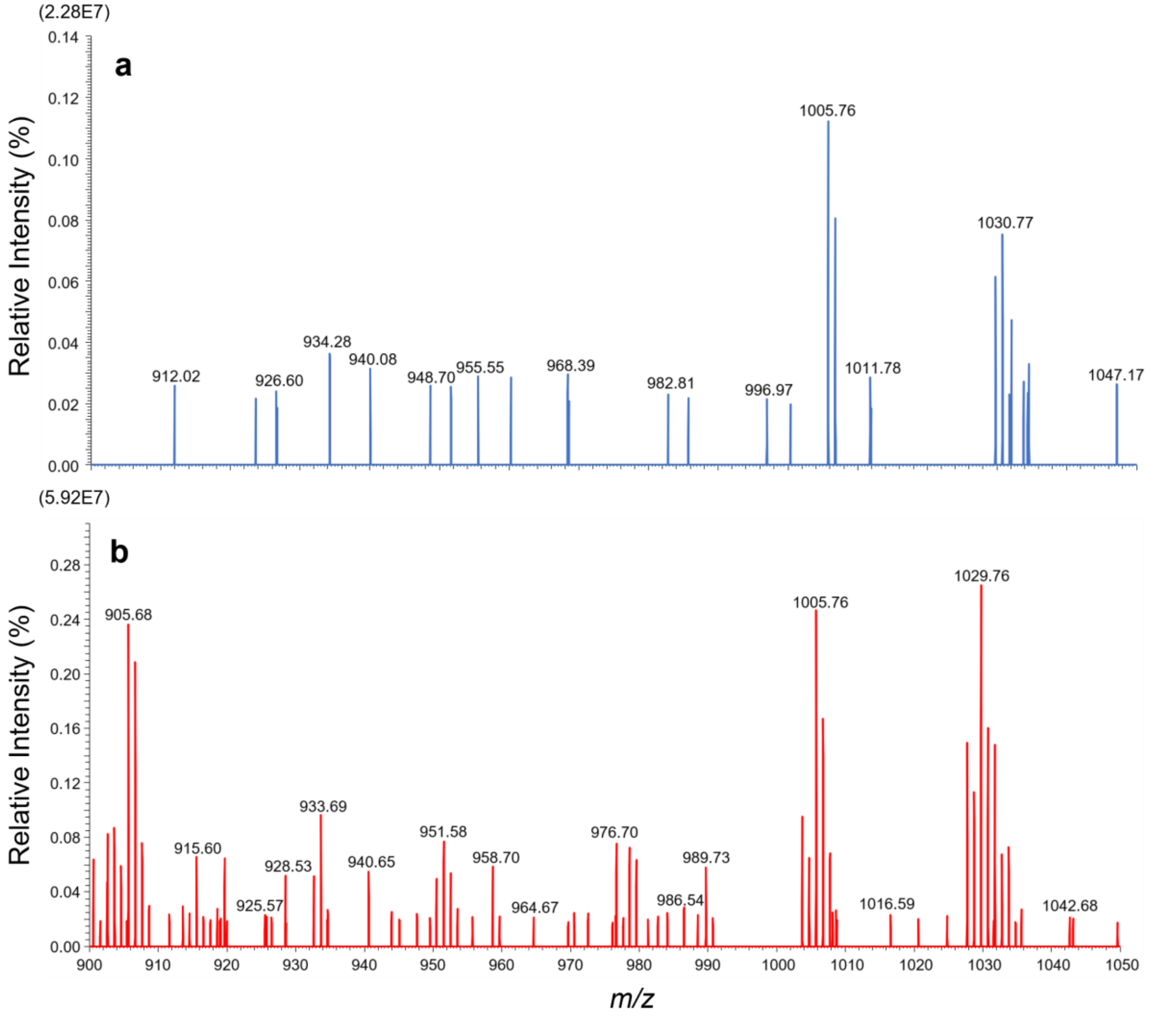

Figure. S5-7. The range of *m/z* 900-1050 in picoPPESI negative ion mode mass spectra obtained from the stalk cells (see Methods) at 3 h in the control (**a**) and after the salt stress treatment (**b**). Voltage: −4 kV; injection sample volume: ca. 200 pL. Data represent repeated experiments with 8–32 stalk cells from 3–6 plants in each treatment. The full mass spectra are shown in Fig. 1.

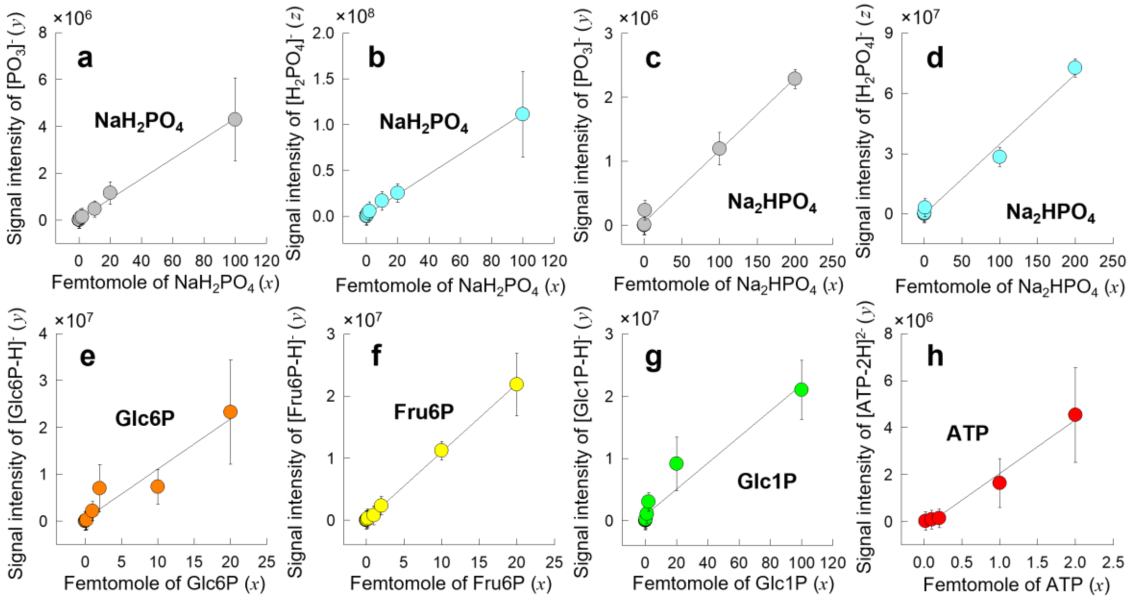

Figure. S6. The relationship between the number of moles of phosphate metabolites and the signal intensity. The relationship between the number of moles of NaH_2PO_4 (x) and signal intensity of $[PO_3]^-$ (y) is $y = 4.28×10^4x + 5.74×10^4$, with $R^2 = 1.00$ (p<0.0001) (**a**), and $[H_2PO_4]^-$ (z) is $z = 1.10×10^6x + 1.80×10^6$, with $R^2 = 1.00$ (p<0.0001) (**b**). The relationship between the number of moles of Na_2HPO_4 (x) and signal intensity of $[PO_3]^-$ (y) is $y = 1.60×10^4x + 1.07×10^3$, with $R^2 = 0.93$ (p<0.0001) (**c**), and $[H_2PO_4]^-$ (z) is $z = 4.84×10^5x + 1.12×10^5$, with $R^2 = 0.99$ (p<0.0001) (**d**). The relationship between the number of moles of glucose 6-phosphate (Glc6P) (x) and signal intensity of $[Glc6P−H]^-$ (y) is $y = 1.07×10^6x + 4.55×10^5$, with $R^2 = 0.93$ (p<0.0001) (**e**). The relationship between the number of moles of fructose 6-phosphate (Fru6P) (x) and signal intensity of $[Fru6P\text{-}H]^-$ (y) is $y = 1.10×10^6x + 6.00×10^3$, with $R^2 = 1.00$ (p<0.0001) (**f**). The relationship between the number of moles of glucose 1-phosphate (Glc1P) (x) and signal intensity of $[Glc1P−H]^-$ (y) is $y = 2.10×10^5x + 8.78×10^5$, with $R^2 = 0.94$ (p<0.0001) (**g**). The relationship between the number of moles of adenosine 5’-triphosphate (ATP) (x) and signal intensity of $[ATP−2H]^{2-}$ (y) is $y = 2.28×10^6x − 2.33×10^5$, with $R^2 = 0.98$ (p<0.0001) (**h**). Data are means±SEs for 3-9 sampling points. Negative ion mode; solvent: water, LC/MS grade (Thermo Scientific); voltage: −4 kV; injection sample volume: ca. 200 pL. Creating the scatter plots and linear regression were performed in SigmaPlot 13.0.

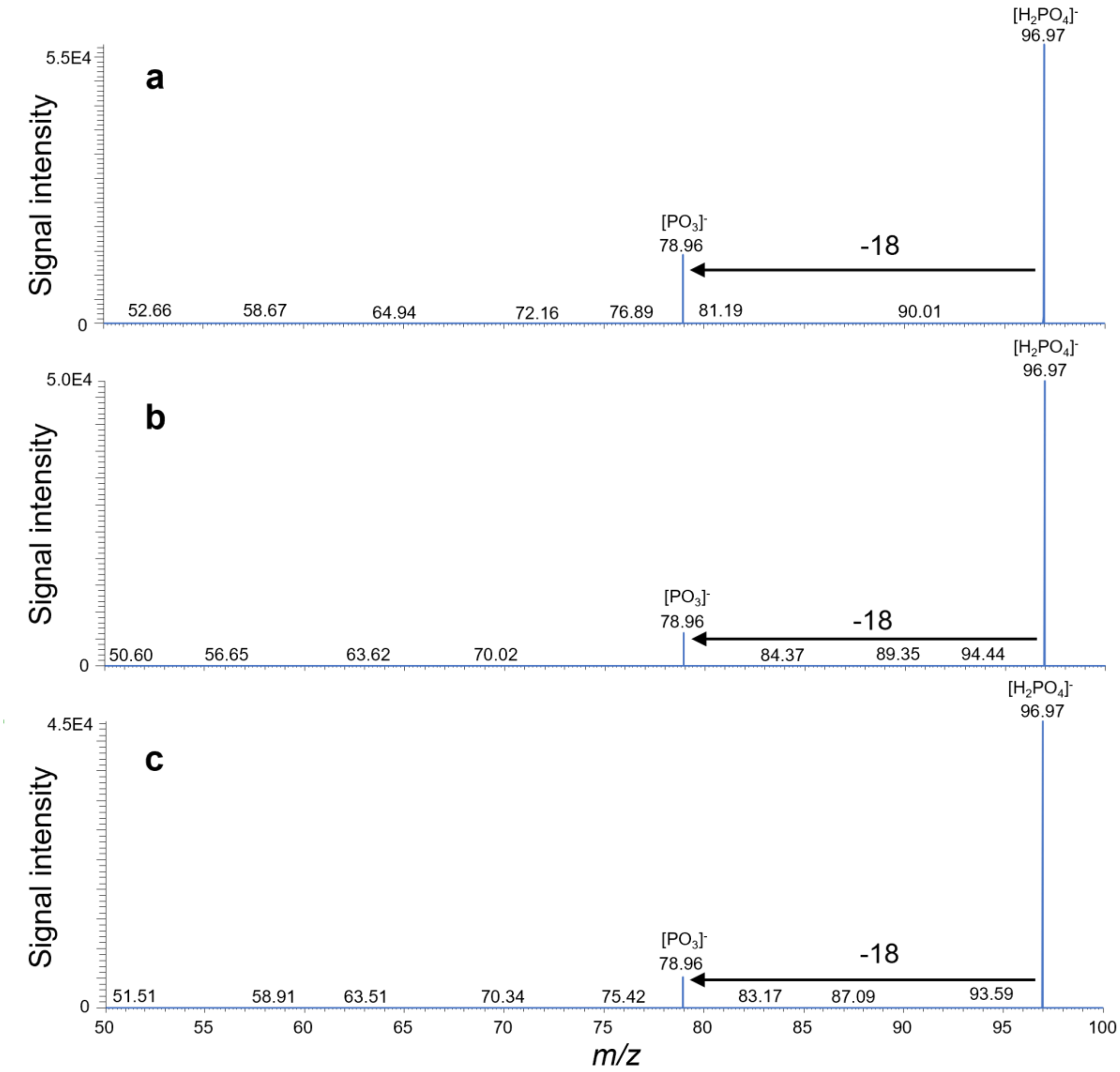

Figure. S7. PicoPPESI-MS/MS spectra of the peak at *m/z* 96.97 from 1.0 mM NaH_2PO_4 (**a**), 1.0 mM Na_2HPO_4 (**b**), and the stalk cell sap (**c**) (see Methods). Precursor ion and the selector gate range was *m/z* 96.97±0.5. The normalized collision energy was set to 0 or 15%, and collision-induced dissociation (CID) fragmentation ions were detected in the orbitrap at a resolution setting of 60,000. Negative ion mode; solvent: water, LC/MS grade (Thermo Scientific); voltage: −4 kV; injection sample volume: ca. 200 pL.

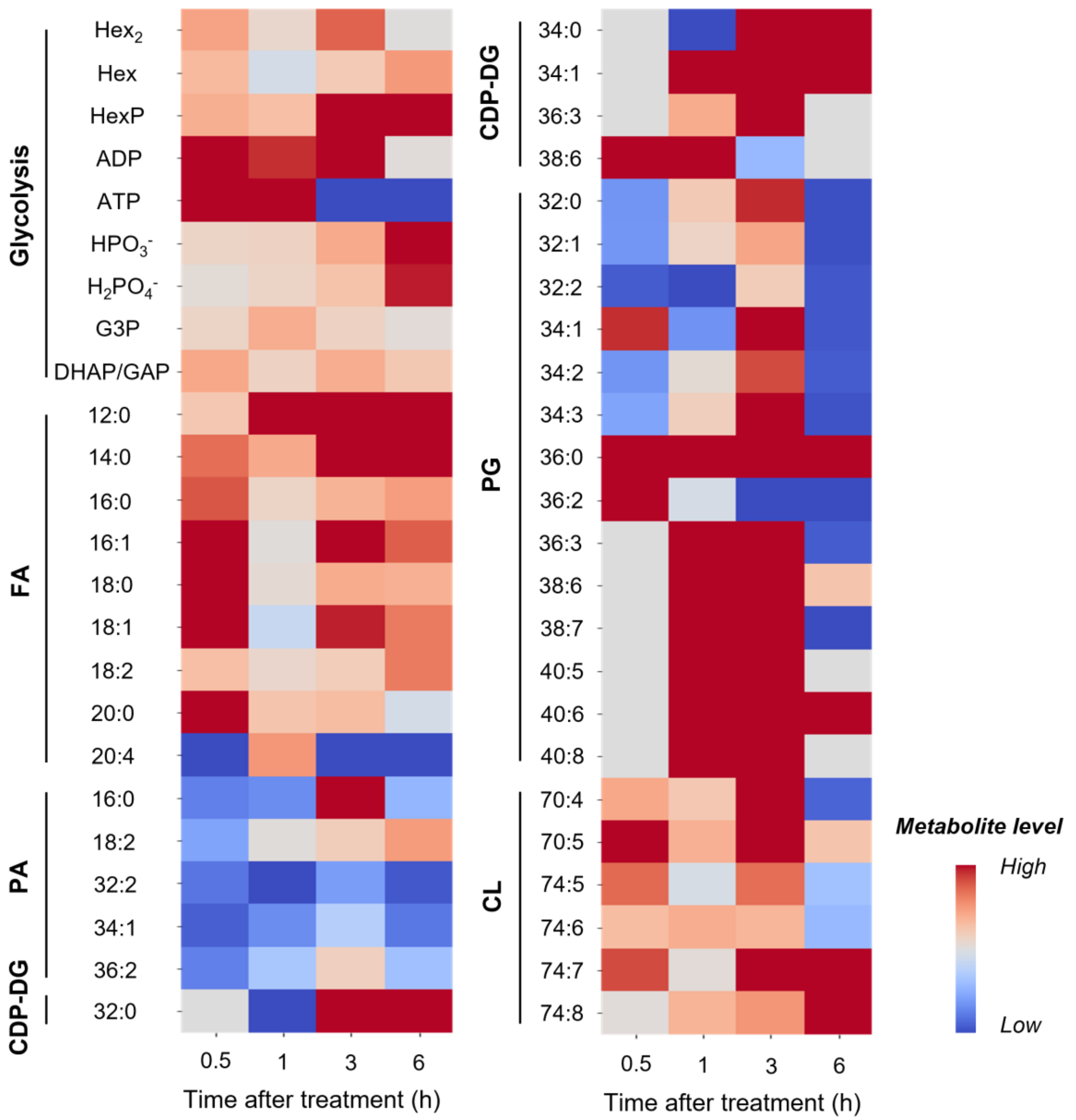

Figure. S8. Changes in cardiolipin (CL) biosynthesis-related metabolites in the stalk cells under salt stress (Ψ_o^{stress} = −0.14 MPa). The level of each metabolite was normalized by baseline (control, Ψ_o^{cont} = −0.05 MPa). Negative ion mode; voltage: −4 kV; injection sample volume: ca. 200 pL. Data were obtained from 8-32 stalk cells in 3-6 plants in each treatment. Hex: hexose; HexP: hexose phosphate; G3P: glycerol-3-phosphate; DHAP: dihydroxyacetone phosphate; GAP: glyceraldehyde 3-phosphate; FA: fatty acid; PA: phosphatidic acid; CDP-DG: cytidine diphosphate diacylglycerol; PG: phosphatidylglycerol. A heatmap was generated with Python 3.9.1.

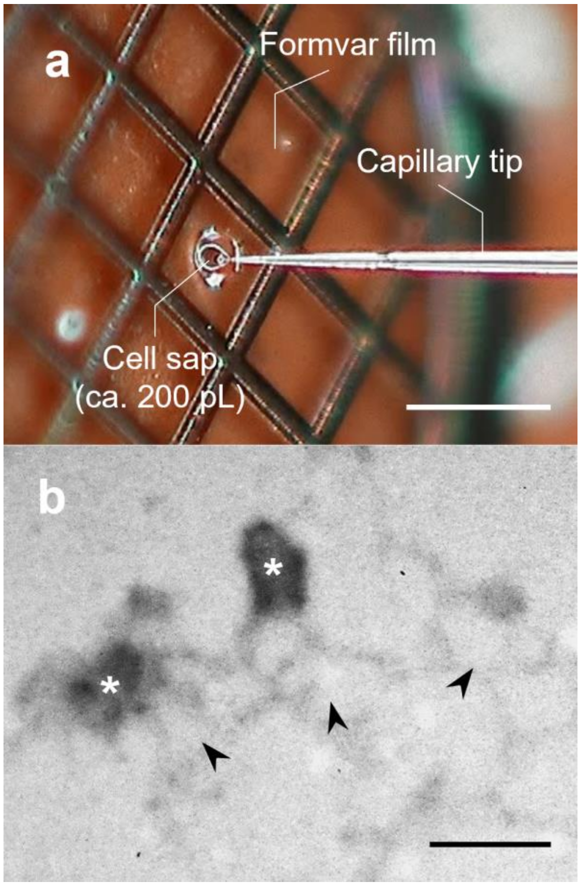

Figure. S9. Stalk cell sap loading onto the formvar film placed on the transmission electron microscopy (TEM) grid by using a cell pressure-probe (CPP) (**a**) and TEM image of mitochondria (asterisk) and membrane-like structures (arrowhead) observed after sampling directly with the CPP (**b**). Bars = 250 μm (**a**) and 1.0 μm (**b**).

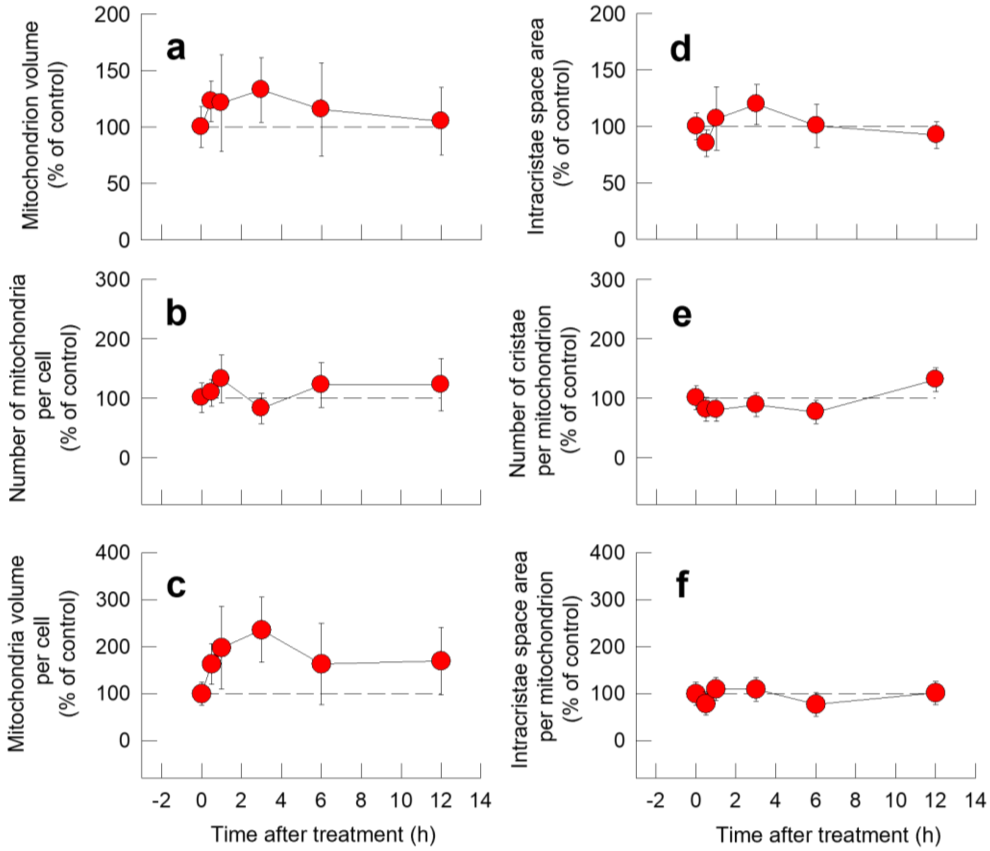

Figure. S10. Changes in mitochondria and the internal structures of stalk cells (see Methods) under the salt stress condtions. Time-course of changes in mitochondrion volume (**a**), number of mitochondria per cell (**b**), total mitochondria volume per cell (**c**), intracrista space area (**d**), the number of cristae per mitochondrion (**e**), and intracrista space area per mitochondrion (**f**). Data are means±SE for 5-12 cells from 3-6 plants in each treatment. Line graphs were created with Sigmaplot 13.0.

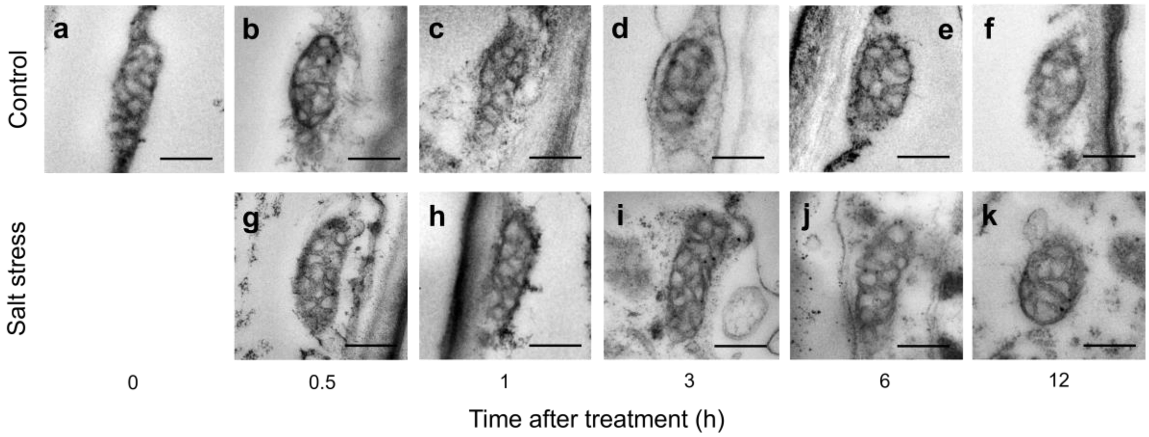

Figure. S11. Time-course of changes in mitochondrial internal structures under the salt stress conditions. Data are representative of repeated experiments with 94-315 cristae in 16-44 mitochondria collected from 3-6 plants in each treatment. Bars = 500 nm.

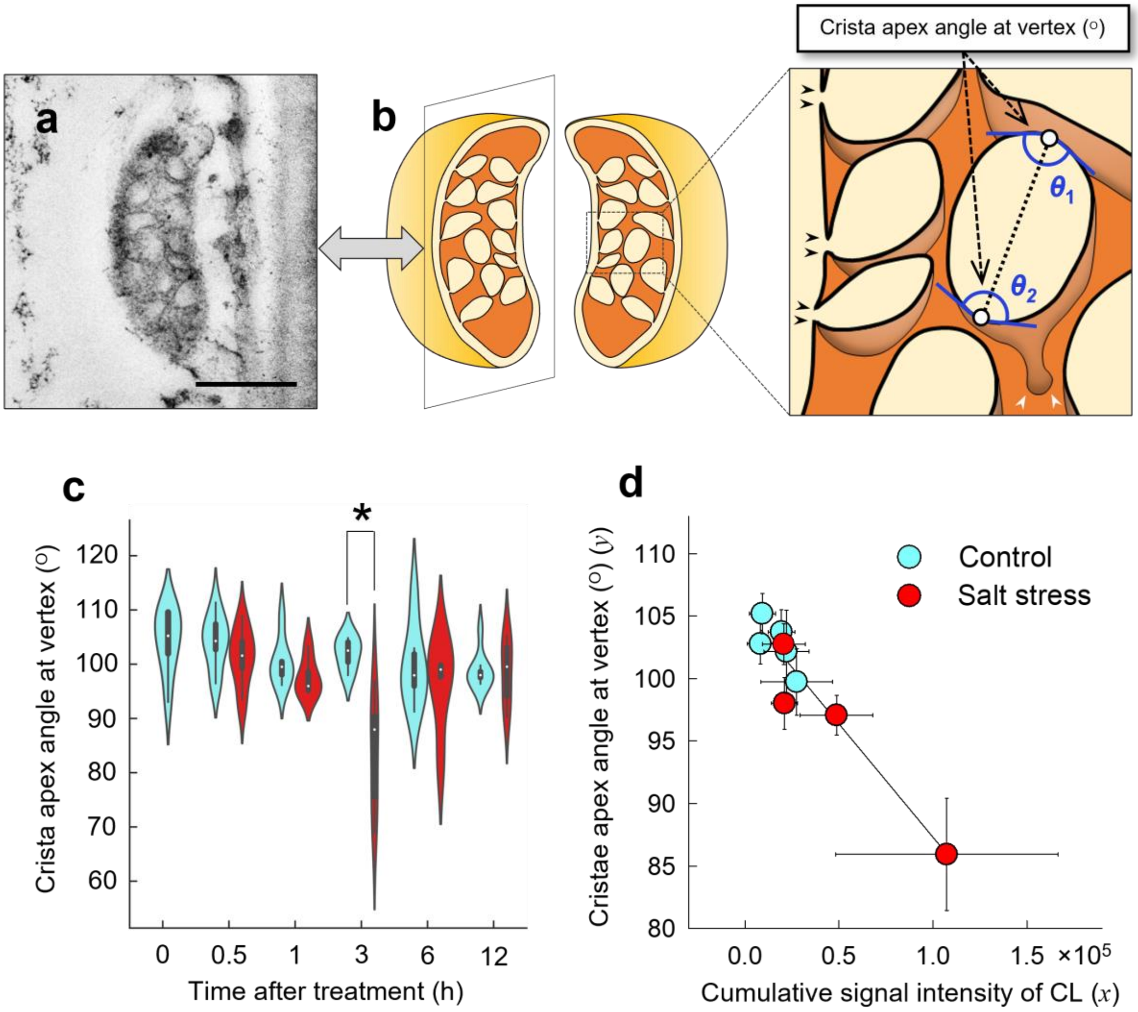

Figure. S12. Morphological changes in crista apex angle (θ) in mitochondria and the internal structures of the stalk cells under salt stress conditions. Longitudinal section of the mitochondrial internal structures and the cristae junction model (**a** and **b**). Bar = 500 nm (**a**). θ of the mitochondria (**c**). Cyan and red violin plots indicate the control and salt stress treatment, and the data were obtained from 94-315 cristae in 16-44 mitochondria in 3-6 plants in each treatment. Significant difference at the 0.01 probability levels by *t*-test is indicated with *. Regression of θ and cumulative signal intensity of cardiolipin (CL) (**d**), and data are means±SEs for 8-32 stalk cells from 3-6 plants. The regression line between the cumulative signal intensity of CL (x) and θ (z) is $z = -18.12 \times 10^{-5}x + 105.43$, with $R^2 = 0.92$ ($p<0.0001$). The violin plot in **c** was created with Python 3.9.1. In **d**, creating a scatter plot and the linear regression were performed in SigmaPlot 13.0.

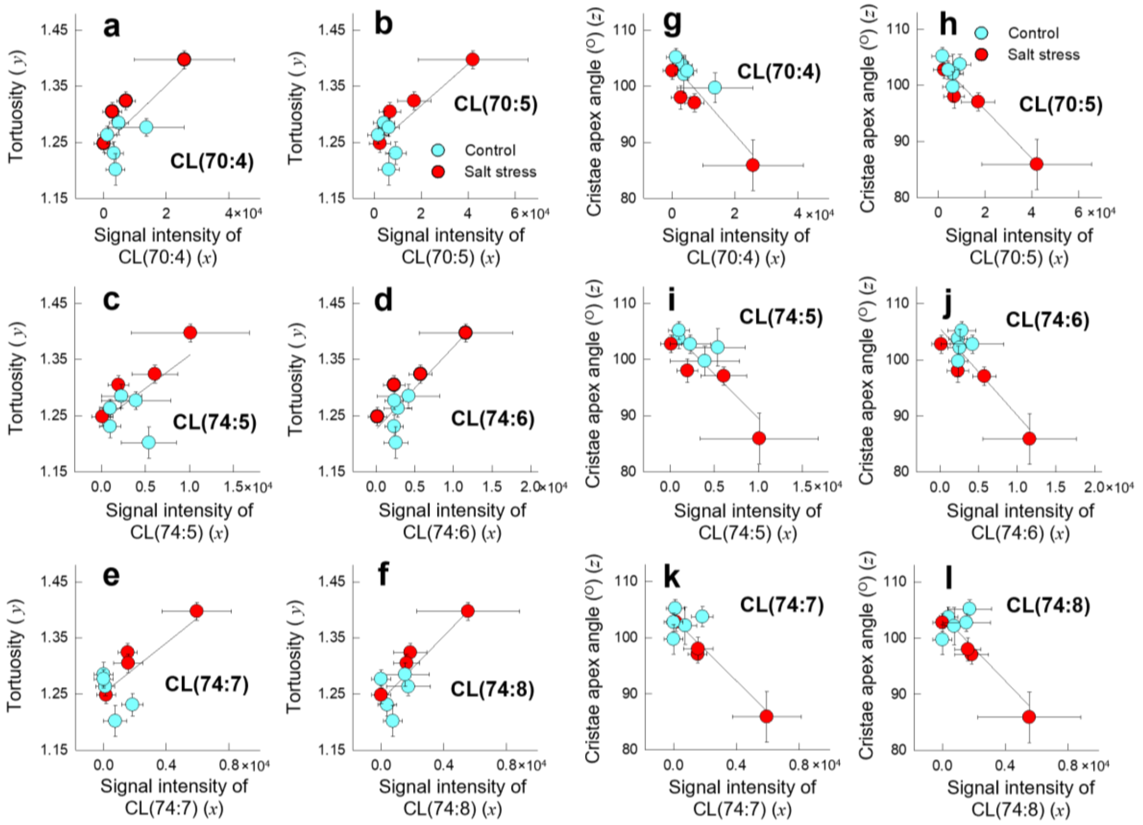

Figure. S13. The relationship between mitochondrial tortuosity (τ) or crista apex angle (θ) (see Materials) and signal intensity of cardiolipin (CL) in the stalk cells detected by picoPPESI-MS. The relationship between the signal intensity of CL (x) and τ (y) in CL(70:4) is $y = 0.05\times10^{-4}x + 1.24$, with $R^2 = 0.58$ (p<0.05) (**a**), CL(70:5) is $y = 0.04\times10^{-4}x + 1.24$, with $R^2 = 0.66$ (p>0.01) (**b**), CL(74:5) is $y = 0.12\times10^{-4}x + 1.24$, with $R^2 = 0.44$ ($p = 0.05$) (**c**), CL(74:6) is $y = 0.15\times10^{-4}x + 1.23$, with $R^2 = 0.71$ (p<0.005) (**d**), CL(74:7) is $y = 0.22\times10^{-4}x + 1.25$, with $R^2 = 0.54$ (p<0.05) (**e**), and CL(74:8) is $y = 0.29\times10^{-4}x + 1.24$, with $R^2 = 0.72$ (p<0.005) (**f**). The relationship between the signal intensity of CL (x) and θ (z) in CL(70:4) is $z = -6.37\times10^{-4}x + 104.13$, with $R^2 = 0.79$ (p<0.005) (**g**), CL(70:5) is $z = -4.35\times10^{-4}x + 104.36$, with $R^2 = 0.89$ (p<0.0005) (**h**), CL(74:5) is $z = -15.65\times10^{-4}x + 105.58$, with $R^2 = 0.74$ (p<0.005) (**i**), CL(74:6) is $z = -15.44\times10^{-4}x + 105.55$, with $R^2 = 0.77$ (p<0.005) (**j**), CL(74:7) is $z = -27.56\times10^{-4}x + 103.35$, with $R^2 = 0.80$ (p<0.005) (**k**), and CL(74:8) is $y = -29.05\times10^{-4}x + 104.04$, with $R^2 = 0.71$ (p<0.005) (**l**). Negative ion mode; voltage: −4 kV; injection sample volume: ca. 200 pL. Data are means±SEs for 8-32 stalk cells from 3-6 plants. Creating the scatter plots and linear regression were performed in SigmaPlot 13.0.

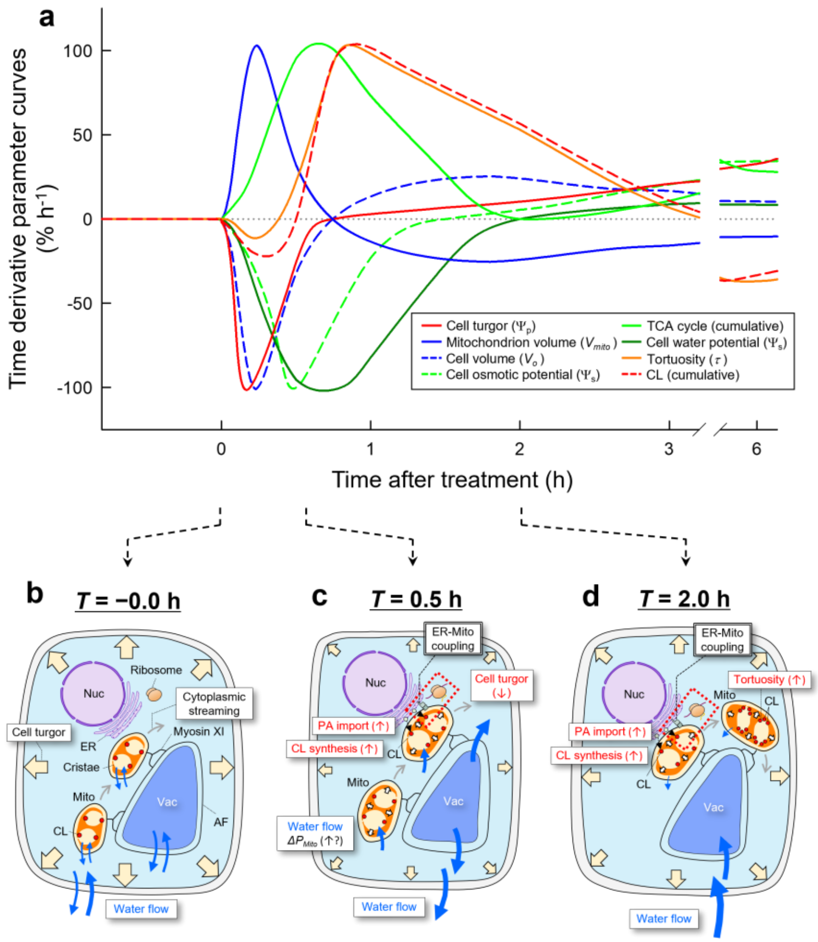

Figure. S14. Time derivative parameter curves of cell turgor (Ψ_p), cell osmotic potential (Ψ_s), cell water potential (Ψ_w), cell volume (V_o), mitochondrion volume (V_{Mito}), the cumulative signal intensities of tricarboxylic acid (TCA) cycle metabolites and cardiolipins (CLs), and cristae tortuosity (τ) (**a**). All parameters were scaled by setting the maximum/minimum absolute value of the change to be +100/−100%, and then the time derivative parameter curves were generated. Diagrams illustrating the cellular events at T = −0.0 h (**b**), 0.5 h (**c**), and 1.5 h (**d**) after treatment based on the data (see Discussion). Red dotted squares in **b-d** correspond to the vicinity of endoplasmic reticulum (ER)-mitochondrion connection (see the expanded schematic diagrams shown in Fig. 5). To simplify schematics, two mitochondria were shown, and other organelles, such as peroxisomes and Goldi body, have been removed. And, the size of vacuoles is drawn to be much smaller than the real situation. Nuc: nucleus; Mito: mitochondria; Vac: vacuole; AF: actin filament(s); PA: phosphatidic acid. The graph was created with Sigmaplot 13.0.

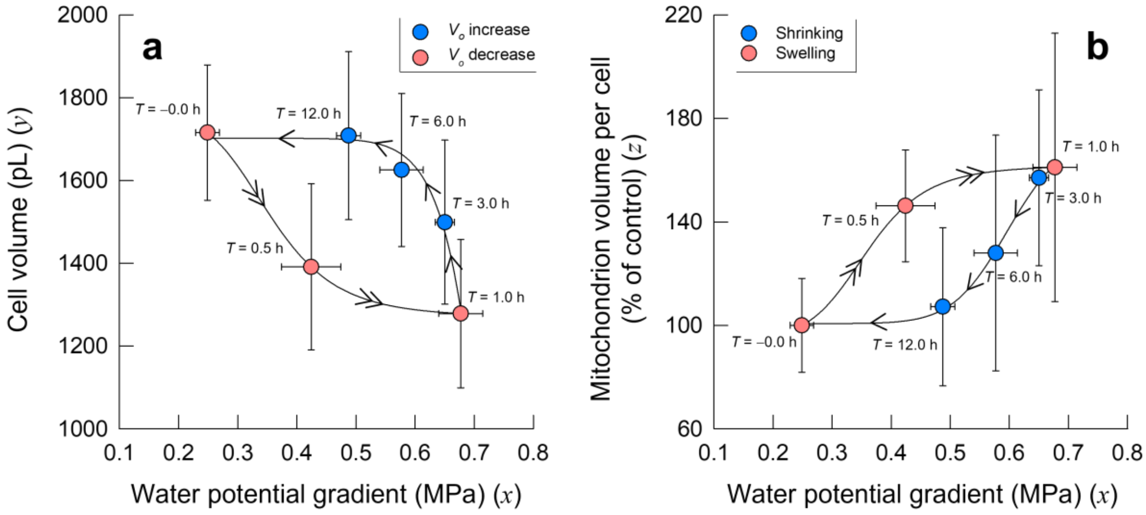

Figure. S15. Hysteresis loops for the volumetric responses of tomato trichome stalk cells (V_o) and their mitochondrial volume at salt stress. Changes in the V_o (**a**) and the mitochondrion volume per cell volume (**b**) as a function of the water potential gradient ($\Psi_o - \Psi_w$, xylem water potential – stalk cell water potential). The values in each figure were adopted from Fig. 1b, e, and g. The regression line between water potential gradient (x) and decreasing V_o (y_1) is $y_1 = 496.75x^{-6.19}/(0.35^{-6.19} + x^{-6.19}) + 1269.63$, with $R^2 = 1.00$ ($p = 1.000$), and increasing V_o (y_2) is $y_2 = 7.65 \times 10^5 x^{-14.87}/(1.12^{-14.87} + x^{-14.87}) - 7.63 \times 10^5$, with $R^2 = 0.98$ ($p = 1.000$) (**a**). The regression line between the water potential gradient (x) and mitochondrion volume at swelling (z_1) is $z_1 = 66.30x^{7.12}/(0.36^{7.12} + x^{7.12}) + 95.50$, with $R^2 = 1.00$ ($p = 1.000$), and mitochondrion volume at shrinking (z_2) is $z_2 = 76.63x^{12.12}/(0.60^{12.12} + x^{12.12}) + 100.67$, with $R^2 = 1.00$ ($p = 0.138$) (**b**). In **a** and **b**, T indicates the time after salt stress treatment, and each regression line fits by Hill equation and is shown in black line. Double and single arrows indicate V_o reduction process with mitochondrial swelling from T = 0.0 to 1.0 h after salt stress treatment and V_o increase process with mitochondrial shrinking from T = 1.0 to 12.0 h after the treatment, respectively. Creating the scatter plots and fitting nonlinear curve were performed in SigmaPlot 13.0.

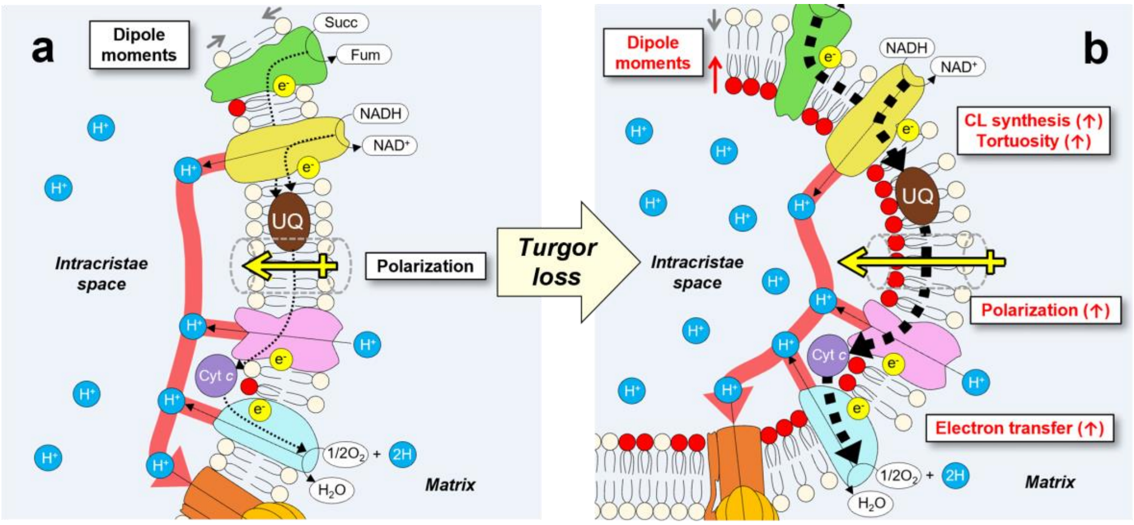

c

Monolayer	Molecular area (nm^2)	Surface potential (mV)	Total dipole moment ($\times 10^{-30}$ C m)	References
PA	0.61	+250	+1.34	[1] Beitinger et al. (1989) *Biochim Biophys Acta* **984**, 293-300 [2] Lintonen et al. (2014) *Anal. Chem.* **86**, 9662-9669
PS	0.42	+329	+1.23	[1] Beitinger et al. (1989) *Biochim Biophys Acta* **984**, 293-300 [2 Lintonen et al. (2014) *Anal. Chem.* **86**, 9662-9669
PE	0.41	+599	+2.16	[1] Beitinger et al. (1989) *Biochim Biophys Acta* **984**, 293-300 [2] Lintonen et al. (2014) *Anal. Chem.* **86**, 9662-9669
PC	0.69	+420	+2.56	[1] Beitinger et al. (1989) *Biochim Biophys Acta* **984**, 293-300 [2] Lintonen et al. (2014) *Anal. Chem.* **86**, 9662-9669
CL	1.29	+326	+3.67	[1] Beitinger et al. (1989) *Biochim Biophys Acta* **984**, 293-300 [3] Sennato et al. (2005) *J. Phys. Chem. B* **109**, 15950-15957

Figure. S16. Morphological response of crista membranes by cardiolipin (CL) to salt stress and polarization by different dipole moments of each phospholipid. CL synthesis induced by turgor loss causes CL-associated crista membrane modifications (**a** and **b**). CL has a different total dipole moment and surface potential than diacylphospholipids (**c**). The dipole density due to crista membrane curvature formation enhances the net polarization, which streamlines electron transfer, and proton (H^+) transfer by respiratory chain complexes. PA: phosphatidic acid; PS: phosphatidylserine; PE: phosphatidylethanolamine; PC: Phosphatidylcholine.

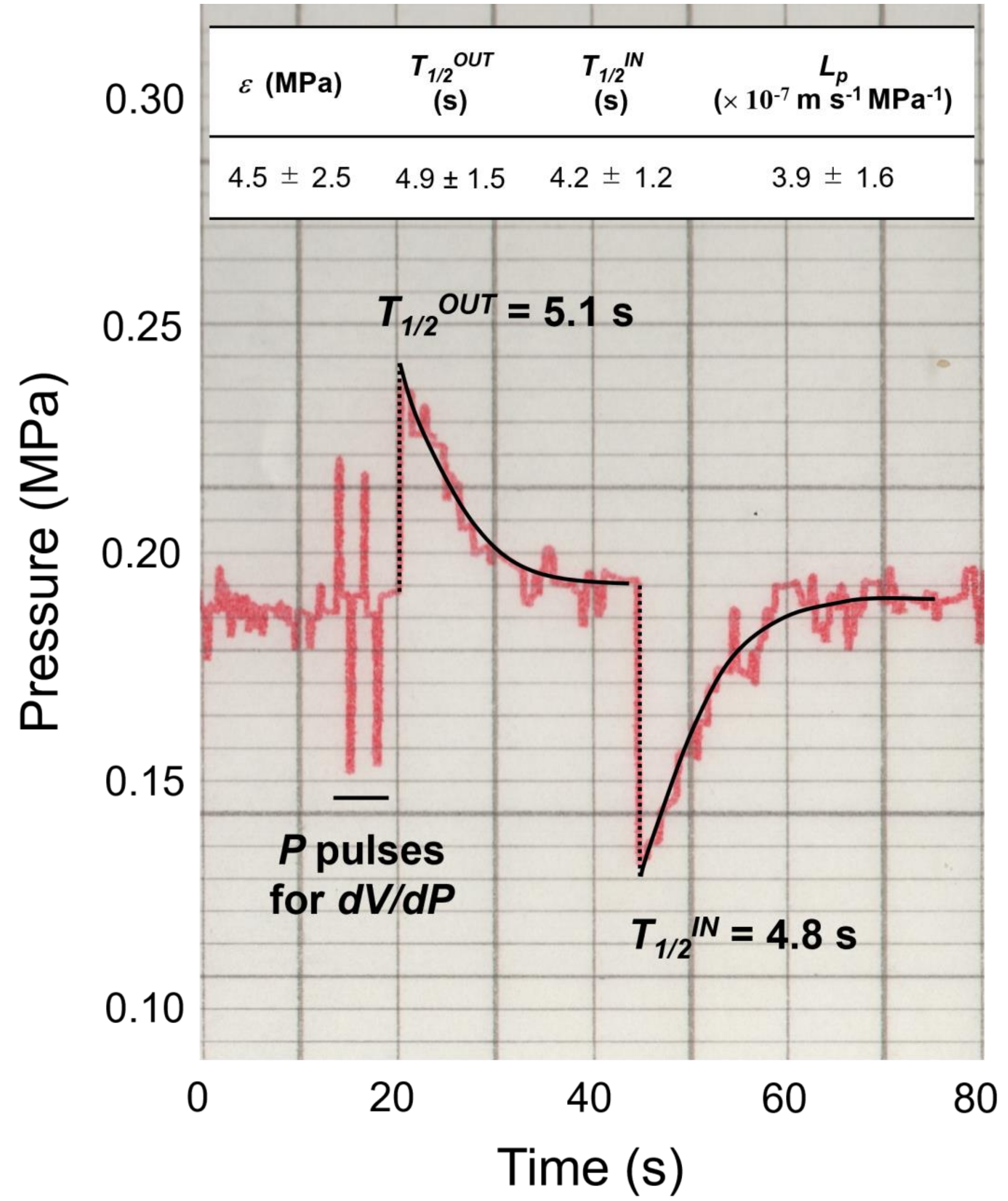

ε (MPa)	$T_{1/2}^{OUT}$ (s)	$T_{1/2}^{IN}$ (s)	L_p ($\times 10^{-7}$ m s^{-1} MPa^{-1})
4.5 ± 2.5	4.9 ± 1.5	4.2 ± 1.2	3.9 ± 1.6

Figure S17. Cell wall elastic modulus (ε), half time ($T_{1/2}$), and hydraulic conductivity (L_p) of the stalk cells (see Methods). $T_{1/2}$ for water permeability in the control cells was individually determined at exosmotic ($T_{1/2}^{OUT}$) or endosmotic flows ($T_{1/2}^{IN}$). Data shown in the table are means±SD for 6-8 cells from 3 plants.

References

1 Beitinger, H., Vogel, V., Möbius, D. & Rahmann, H. Surface potentials and electric dipole moments of ganglioside and phospholipid bilayers: contribution of the polar headgroup at the water/lipid interface. *Biochim. Biophys. Acta Biomembr.* **984**, 293-300, https://doi.org/10.1016/0005-2736(89)90296-4 (1989).

2 Lintonen, T. P. et al. Differential mobility spectrometry-driven shotgun lipidomics. *Anal. Chem.* **86**, 9662-9669, https://doi.org/10.1021/ac5021744 (2014).

3 Sennato, S. et al. Evidence of domain formation in cardiolipin-glycerophospholipid mixed monolayers. A thermodynamic and AFM study. *J. Phys. Chem. B* **109**, 15950-15957, https://doi.org/10.1021/jp051893q (2005).
